# Age Trajectories of the Structural Connectome in Child and Adolescent Offspring of Individuals With Bipolar Disorder or Schizophrenia

**DOI:** 10.1016/j.bpsgos.2024.100336

**Published:** 2024-05-28

**Authors:** Simon R. Poortman, Marjolein E.A. Barendse, Nikita Setiaman, Martijn P. van den Heuvel, Siemon C. de Lange, Manon H.J. Hillegers, Neeltje E.M. van Haren

**Affiliations:** a Department of Child and Adolescent Psychiatry/Psychology, Erasmus University Medical Center, Sophia Children’s Hospital, Rotterdam, the Netherlands; b Department of Psychiatry, University Medical Center Utrecht Brain Center, Utrecht, the Netherlands; c Department of Complex Trait Genetics, Center for Neurogenomics and Cognitive Research, Vrije Universiteit Amsterdam, Amsterdam, the Netherlands; d Department of Child Psychiatry, Amsterdam University Medical Center, Amsterdam Neuroscience, Amsterdam, the Netherlands; e Department of Sleep and Cognition, Netherlands Institute for Neuroscience, an institute of the Royal Netherlands Academy of Arts and Sciences, Amsterdam, the Netherlands

**Keywords:** Bipolar disorder, Development, High familial risk, Offspring, Schizophrenia, White matter

## Abstract

**Background:**

Offspring of parents with severe mental illness (e.g., bipolar disorder or schizophrenia) are at elevated risk of developing psychiatric illness owing to both genetic predisposition and increased burden of environmental stress. Emerging evidence indicates a disruption of brain network connectivity in young offspring of patients with bipolar disorder and schizophrenia, but the age trajectories of these brain networks in this high-familial-risk population remain to be elucidated.

**Methods:**

A total of 271 T1-weighted and diffusion-weighted scans were obtained from 174 offspring of at least 1 parent diagnosed with bipolar disorder (*n* = 74) or schizophrenia (*n* = 51) and offspring of parents without severe mental illness (*n* = 49). The age range was 8 to 23 years; 97 offspring underwent 2 scans. Anatomical brain networks were reconstructed into structural connectivity matrices. Network analysis was performed to investigate anatomical brain connectivity.

**Results:**

Offspring of parents with schizophrenia had differential trajectories of connectivity strength and clustering compared with offspring of parents with bipolar disorder and parents without severe mental illness, of global efficiency compared with offspring of parents without severe mental illness, and of local connectivity compared with offspring of parents with bipolar disorder.

**Conclusions:**

The findings of this study suggest that familial high risk of schizophrenia is related to deviations in age trajectories of global structural connectome properties and local connectivity strength.

## Plain Language Summary

In this article, we show that child and adolescent offspring of parents with schizophrenia had different patterns in the development of their brain’s structural connections compared with offspring of parents with bipolar disorder and offspring of parents without these conditions. The findings of this long-term study indicate that having a family history of schizophrenia is associated with changes over time during adolescence in the overall organization of the brain’s structural network.

Offspring of parents with severe mental illness are particularly prone to developing psychopathology (1,2). Specifically, offspring of parents with bipolar disorder (BDo) or schizophrenia (SZo) are at a respective 2.1- and 2.6-fold increased risk of developing at least 1 psychiatric disorder compared with offspring of parents without these mental illnesses (Co) (3, 4, 5, 6). Elucidating the putatively divergent neurobiological networks in these high-familial-risk offspring may help disentangle the developmental roots of the brain anomalies seen in manifested illness (7, 8, 9, 10), thereby shedding light on the etiological mechanisms that underlie risk of intergenerational transmission of mood-psychosis disorders. Adolescence is the ideal window to examine this because it is associated with emergent psychopathology (11,12) and a period of substantial development of the brain’s white matter (13,14).

A growing body of literature has demonstrated disruptions of the connectome—the comprehensive network of the brain’s structural connections—in individuals with bipolar disorder (15, 16, 17, 18, 19, 20, 21, 22, 23) and schizophrenia (24, 25, 26, 27, 28, 29, 30, 31, 32), as well as their siblings (33) and offspring (15,34, 35, 36). A recurrent finding in both patient groups (20, 21, 22, 23, 24, 25, 26, 27,30) and their relatives (33,34) has been the aberrant connectivity of the brain network’s “rich club” (RC), a coherent constellation of highly interconnected hubs reputed to act as the backbone for global brain integration (37, 38, 39). Most of the studies conducted to date are cross-sectional and thus provide no insight into the developmental trajectory of this central system of brain connectivity. A longitudinal study design with child and adolescent offspring at high familial risk enables the estimation of the connectome trajectories that precede the peak age of onset of severe mental illness. Importantly, such trajectories may reveal the age at which possible deviations start to emerge in high-familial-risk offspring. If the trajectories are lower or higher across the entire included age range, divergence begins around early childhood. However, if deviations first become apparent during adolescence, this may be a period of interest for studying interactions with processes that occur during this phase (e.g., puberty, social interactions, substance use) or it may be a target window for intervention or preventive strategies.

Two cohort studies have examined white matter changes over time in individuals at familial risk of bipolar disorder (36,40, 41, 42). The Scottish Bipolar Family Study found no differences between individuals at familial risk of bipolar disorder and control individuals in 2-year trajectories of fractional anisotropy (FA) (40). Similarly, the New South Wales study reported no differences in the prevalence of white matter hyperintensities over a 2-year period (41). In contrast, in their recent tractography study, high-familial-risk individuals presented an increase of FA in the right hippocampal cingulum over time relative to control individuals (42) and, in their connectome study, diminishing connectivity of a subnetwork comprising various cortical and subcortical regions, whereas connections in this network strengthened in control individuals over a 2-year period (36). However, no studies have been conducted of age-related trajectories of white matter or structural connectivity in individuals at familial risk of schizophrenia. The joint investigation of BDo or SZo may reveal transdiagnostic and pathognomonic risk and resilience factors related to connectome development.

In this prospective cross-disorder study, we aimed to compare trajectories of anatomical brain network metrics of child and adolescent BDo or SZo and Co to discover disorder-specific deviations from normal development.

## Methods and Materials

### Participants

The current study is part of the longitudinal Dutch Bipolar and Schizophrenia Offspring study. After exclusions for scan quality and other reasons (Supplemental Methods and Table S1), the current sample includes a total of 271 magnetic resonance imaging (MRI) brain scans of 174 participants (74 BDo, 51 SZo, and 49 Co) from 120 families (Table S2). At time point 1, 126 individuals ages between 8 and 18 years comprised 51 BDo, 35 SZo, and 40 Co. At time point 2, 145 participants ages between 11 and 23 years included 64 BDo, 39 SZo, and 42 Co. A total of 97 individuals (41 BDo, 23 SZo, and 33 Co) were scanned at both time points, with 2.2 to 5.9 years between assessments (mean = 3.8 years) (Table 1 and Table S3). Despite the hybrid nature of the sample (including both cross-sectional and longitudinal data), we use the term longitudinal to describe the study design, and we used all available data to reduce bias and improve power. Two scanners were used (Supplemental Methods; Tables S4 and S5). Participants were considered to be at familial risk if they had at least 1 first- or 2 second-degree relatives with bipolar disorder or schizophrenia. Given that the vast majority of the final sample comprises offspring (169 of 174 participants; 97%) and for the sake of readability, we decided to use the term offspring. In the final sample, 7 BDo (from 4 families) had 2 parents with bipolar disorder and 3 SZo (from 2 families) had 1 parent with schizophrenia and 1 parent with bipolar disorder; the rest of the high-familial-risk offspring had 1 parent with BDo or SZo. Clinical diagnoses of index parents were confirmed using the Structured Clinical Interview for DSM-IV Axis I disorders (43). Parents of Co were screened for psychopathology with the Mini-International Neuropsychiatric Interview Schedules for Clinical Assessment in Neuropsychiatry (44) followed by Structured Clinical Interview for DSM-IV Axis I disorders in case of reported psychopathology. At time point 1, 96% of the offspring had never used psychotropic medication. At time point 2, this was 83%.

**Table 1:** Demographic and Clinical Characteristics

	BDo		SZo		Co		Main Group Effect				Pairwise, *p* < .05	
	Time Point 1, *n* = 51	Time Point 2, *n* = 64	Time Point 1, *n* = 35	Time Point 2, *n* = 39	Time Point 1, *n* = 40	Time Point 2, *n* = 42	Time Point 1		Time Point 2		Time Point 1	Time Point 2
							*F(df)*	*p*	*F(df)*	*p*		
Age at Scan, Years, Mean (SD)	14.19 (2.57)	17.86 (2.51)	13.54 (2.74)	16.51 (2.89)	13.52 (2.16)	16.89 (2.45)	1.06 (2,123)	.350	3.71 (2,142)	.027^a^	–	BDo > SZo
Sex, Female/Male, *n* (Female %)	24/27 (47%)	30/34 (47%)	21/14 (60%)	30/9 (77%)	20/20 (50%)	21/21 (50%)	–	.516	–	.007^a^	–	SZo > BDo and Co
IQ, Mean (SD)	106.3 (19.6)	104.8 (14.8)	103.3 (18.3)	101.9 (19.2)	116.1 (12.3)	113.7 (13.0)	5.92 (2,123)	.003^a^	6.41 (2,142)	.002^a^	BDo and SZo < Co	BDo and SZo < Co
Scan Interval, Years, Mean (SD)^b^	3.95 (0.71)		3.83 (0.59)		3.70 (1.01)		*F* = 0.84 (2,94), *p* = .436				–	–
DSM-IV Diagnosis, *n* (%)												
No diagnosis	26 (51.0%)	24 (37.5%)	16 (45.7%)	11 (28.2%)	32 (80.0%)	31 (73.8%)	–	.003^a^	–	<.001^a^	BDo and SZo < Co	BDo and SZo < Co
Developmental disorder^c^	8 (15.7%)	18 (28.1%)	7 (20.0%)	15 (38.5%)	1 (2.5%)	5 (11.9%)	–	.040^a^	–	.020^a^	SZo > Co	SZo > Co
Anxiety disorder^d^	5 (9.8%)	10 (15.6%)	6 (17.1%)	9 (23.1%)	2 (5.0%)	3 (7.1%)	–	.242	–	.133	–	–
Mild mood disorder^e^	16 (31.4%)	19 (29.7%)	2 (5.7%)	5 (12.8%)	3 (7.5%)	4 (9.5%)	–	.002^a^	–	.022^a^	BDo > SZo and Co	BDo > Co
Major depressive disorder	1 (2.0%)	7 (10.9%)	5 (14.3%)	7 (17.9%)	0 (0.0%)	0 (0.0%)	–	.007^a^	–	.011^a^	SZo > BDo and Co	BDo and SZo > Co
Manic disorder^f^	2 (3.9%)	2 (3.1%)	0 (0.0%)	1 (2.6%)	0 (0.0%)	0 (0.0%)	–	.337	–	.619	–	–
Psychotic disorder	0 (0.0%)	0 (0.0%)	0 (0.0%)	0 (0.0%)	0 (0.0%)	0 (0.0%)	–	–	–	–	–	–
Substance use disorder^g^	2 (3.9%)	5 (7.8%)	1 (2.9%)	3 (5.1%)	0 (0.0%)	0 (0.0%)	–	.624	–	.177	–	–
Other	7 (13.7%)	14 (21.9%)	8 (22.9%)	8 (20.5%)	4 (10.0%)	5 (11.9%)	–	.307	–	.431	–	–
Psychotropic Medication^h^, *n* (%)	5 (9.8%)	14 (21.9%)	0 (0.0%)	8 (20.5%)	0 (0.0%)	3 (7.1%)	–	.022^a^	–	.106	NS	–

Statistical comparisons were performed using Fisher’s exact test for categorical variables and analyses of variance (Tukey’s test for pairwise comparisons) for continuous variables.

BDo, offspring of parents with bipolar disorder; Co, offspring of control parents; NS, not significant; SZo**,** offspring of parents with schizophrenia.

a *p* < .05.

b Ninety-seven (41 BDo, 23 SZo, and 33 Co) of 174 offspring (56%) were scanned at both time points.

c Developmental disorders include attention-deficit/hyperactivity disorder, autism spectrum disorder (including Asperger syndrome and childhood disintegrative disorder), conduct disorder, disruptive behavior disorder not otherwise specified, and oppositional defiant disorder.

d Anxiety disorders include acute stress disorder, adjustment disorder with anxiety, agoraphobia, anxiety disorder not otherwise specified, generalized anxiety disorder, obsessive-compulsive disorder, panic disorder, posttraumatic stress disorder, separation anxiety disorder, social anxiety disorder, and specific phobia.

e Mild mood disorders include adjustment disorder with depressed mood, depressive disorder not otherwise specified, dysthymic disorder, and mood disorder not otherwise specified.

f Manic disorders include bipolar I and II disorders, bipolar disorder not otherwise specified, cyclothymic disorder, hypomania, and mania.

g Substance use disorders include alcohol abuse, alcohol dependence, alcohol use disorder not otherwise specified, substance abuse, substance dependence, and substance use disorder not otherwise specified.

h Psychotropic medications include antidepressants, antipsychotics, methylphenidate, and mood stabilizers.

Written informed consent was obtained from participants who were older than 12 years and from both parents or legal caregivers for participants who were between 8 and 18 years old. Parents also gave written consent for their own participation. The study was approved by the Medical Ethics Committee of the University Medical Center Utrecht. In a previous study, we reported on the group comparisons of connectome metrics in a subsample scanned at time point 1 that partly overlaps with the current offspring cohort (34) (this analysis was repeated on the current time point 1 sample) (see Supplemental Methods, Supplemental Results, and Table S6). In the current study, we extended our previous work by investigating the change over time in network metrics by estimating the trajectories of network development with increasing age.

### MRI Acquisition and Preprocessing

**Figure 1:**
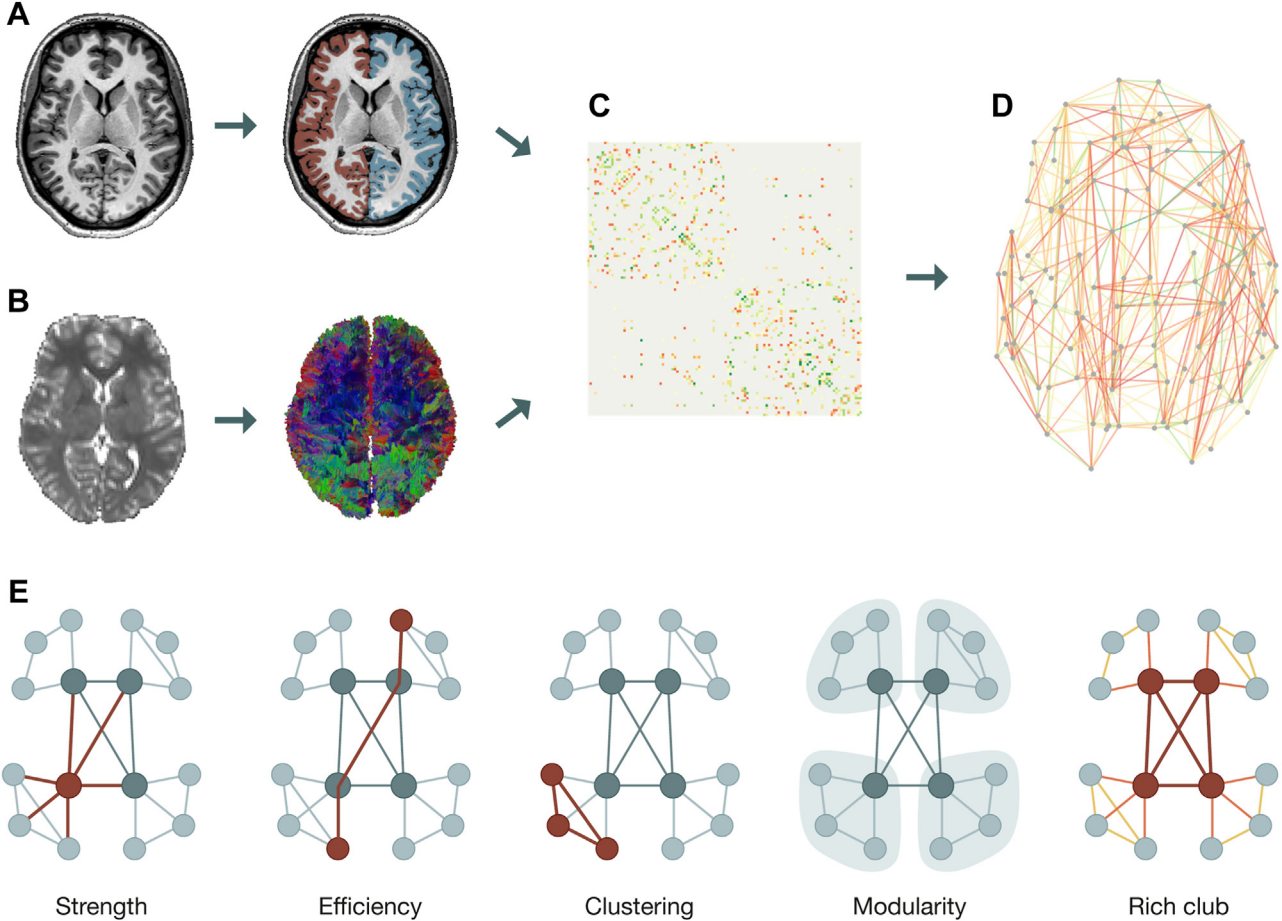
Global processing and analysis steps. **(A)** For each individual participant, a T1-weighted image was used for classification of gray and white matter tissue and parcellation of the cortex into 114 distinct brain regions, which make up the nodes of each individual brain network. **(B)** Diffusion tensor imaging was applied to the diffusion-weighted image of each individual participant. Streamline tractography was performed on the diffusion tensor imaging data to reconstruct the white matter pathways connecting the cortical brain regions. The reconstructed streamlines that interconnect each pair of brain regions form the edges between the nodes of the brain network. Number of streamlines was taken as the weight of each edge. **(C)** A structural connectivity matrix was then obtained for each individual, in which rows and columns denote nodes and entries represent edges (log-transformed for visualization purposes). **(D)** Visual representation of an individual structural brain network derived from the connectivity matrix. **(E)** Schematic illustrations of (from left to right) connectivity strength, global efficiency, clustering, modularity, and rich club measures (red node: hub, gray node: nonhub, red lines: rich club edge, orange lines: feeder edge, yellow lines: local edge).

MRI brain scans were obtained on a Philips 3T Achieva or Philips 3T Ingenia CX scanner (Philips Medical Systems) located at the University Medical Center Utrecht. T1-weighted and diffusion-weighted imaging (DWI) data were (pre)processed using FreeSurfer (version 7.1.1) (45) and the FSL (version 6.0.6) (46). The Connectivity Analysis TOolbox (version 3.2.1) (47) was used to reconstruct structural connectivity from the processed DWI data (Figure 1A, B). Visual quality control was performed by SRP and MEAB at different stages of the process. See Supplemental Methods for a detailed description of the MRI acquisition and (pre)processing procedure and Table S7 for the effects of in-scanner head movement on our main analyses.

### Structural Network Reconstruction

A structural brain network was reconstructed for each individual participant. Each network comprised 114 cortical areas (i.e., nodes)—reflecting a subdivision of the Desikan-Killiany atlas (48,49)—and the reconstructed streamlines between these regions. A connection between 2 nodes (i.e., edge) was included in the network when at least 5 tractography streamlines connected them to each other (50). A structural connectivity matrix containing this network information was created for each participant in which rows and columns represent cortical brain regions, and matrix entries correspond to the weights of the edges (i.e., the number of tractography streamlines [NOS]) (Figures 1C, D). The connectivity strength, global efficiency, clustering coefficient, and modularity of each network were computed to investigate possible differences in the development of overall connectome topology between the 3 groups (51) (Figure 1E) (see Supplemental Methods for a detailed description of each network metric). Because FA values are often used as a marker for white matter integrity in case-control studies (9,10,52), the main analyses were also performed using FA as connection weights (Supplemental Results and Table S8).

### RC Organization

The RC in a network represents a set of highly connected (high-degree) central nodes (i.e., hubs) that are more densely interconnected than would be predicted based on their high degree alone (37, 38, 39). For the main analysis, the hubs comprising the RC were based on previous literature that reported on patients and their relatives as well as the general population (17,30,33,38,39,53,54), including bilateral subregions of the superior frontal gyrus, superior parietal gyrus, insula, and precuneus, accounting for 20 nodes of the total of 114 in each network. This a priori selection of hubs ensured an unbiased selection across the 3 groups. To verify this a priori RC definition, the hubs and RC organization of the 3 groups were compared based on their group-averaged networks (Supplemental Methods). RC analyses were repeated with hubs selected for each group separately based on their group-averaged networks (Supplemental Methods and Supplemental Results).

Categorization of brain regions as hub and nonhub nodes allowed connections to be categorized into 3 classes: RC edges (hub-to-hub connections), feeder edges (hub-to-nonhub connections), and local edges (nonhub-to-nonhub connections) (Figure 1E). RC, feeder, and local connectivity were computed as the sum of the weights of each edge class, leading to 1 value for each edge class for each individual network.

### Statistical Analysis

Statistical analyses were performed using the lme4 package (version 1.1-32) in R (version 4.1.0).

### Age Trajectories of the Structural Connectome

To evaluate group differences in age trajectories of graph metrics, a linear mixed-model analysis was performed to account for 1) the correlation between 2 measurements from the same person, 2) the correlation between individuals from the same family, and 3) missing data, such that individuals scanned at only 1 time point could be included as well, thereby reducing attrition bias. A 3-level model was applied, including measurements within participants and within families. Age was centered around each individual offspring’s mean (i.e., the mean of age at each scan for offspring with 2 visits and age at scan for offspring with 1 visit). Network metrics were modeled as a function of group (SZo, BDo, and Co), age (at each measurement), and the interaction between group and age. As a result, *B* values represent the mean network metric change per year in each group. Potential confounding effects of sex and scanner were corrected for by adding them to the model as fixed effects. Participant ID and family ID were added as random effects (to account for 2 or more relatives from one family), resulting in the following formula: lmer(NetworkMetric ∼ group + age + group × age + sex + scanner + (1|family ID/participant ID)).

### Multiple Comparison Correction

The 7 investigated network metrics demonstrated high levels of correlation (mean [SD] = 0.53 [0.40] after averaging the values for those who were scanned at both time points) (Figure S1), as has been reported in previous studies (55, 56, 57). To control for multiple comparisons while taking the correlation between network metrics into account, a partial Bonferroni-corrected α was computed (58, 59, 60). The outcome values of the 7 NOS-weighted network metrics were scaled by computing their *z* scores. Individuals scanned at both time points had their values averaged first to account for the longitudinal aspect of the study (resulting in 174 values for each metric), and then the mean and standard deviation with which the *z* score was computed were calculated. A principal component analysis was then performed on these *z* scores, and the first 3 components explained 94% of the variance (Table S9). Based on this result, the partial Bonferroni-adjusted α became 0.05/3 = 0.0167. Both the correlation matrix and principal component analysis results are also given for time point 1 and time point 2 data separately, and they produced highly similar results (Table S9 and Figure S1).

### Sensitivity Analyses

To explore whether IQ, psychotropic medication use, or presence of any lifetime psychiatric diagnosis explained the findings (see Supplemental Methods for the reasoning behind the chosen variables), the main analysis was repeated once with IQ, once with medication use (yes/no), and once with lifetime DSM-IV Axis I diagnosis (yes/no) added as a fixed effect.

## Results

### Demographic and Clinical Characteristics

An overview of the demographic and clinical characteristics of each group is provided in Table 1. Because there were group differences in age, sex, IQ, and psychopathology, we included these as control variables (age and sex in all models, IQ and psychopathology in sensitivity analyses).

### Age Trajectories of the Structural Connectome

#### Structural Connectome Topology

**Figure 2:**
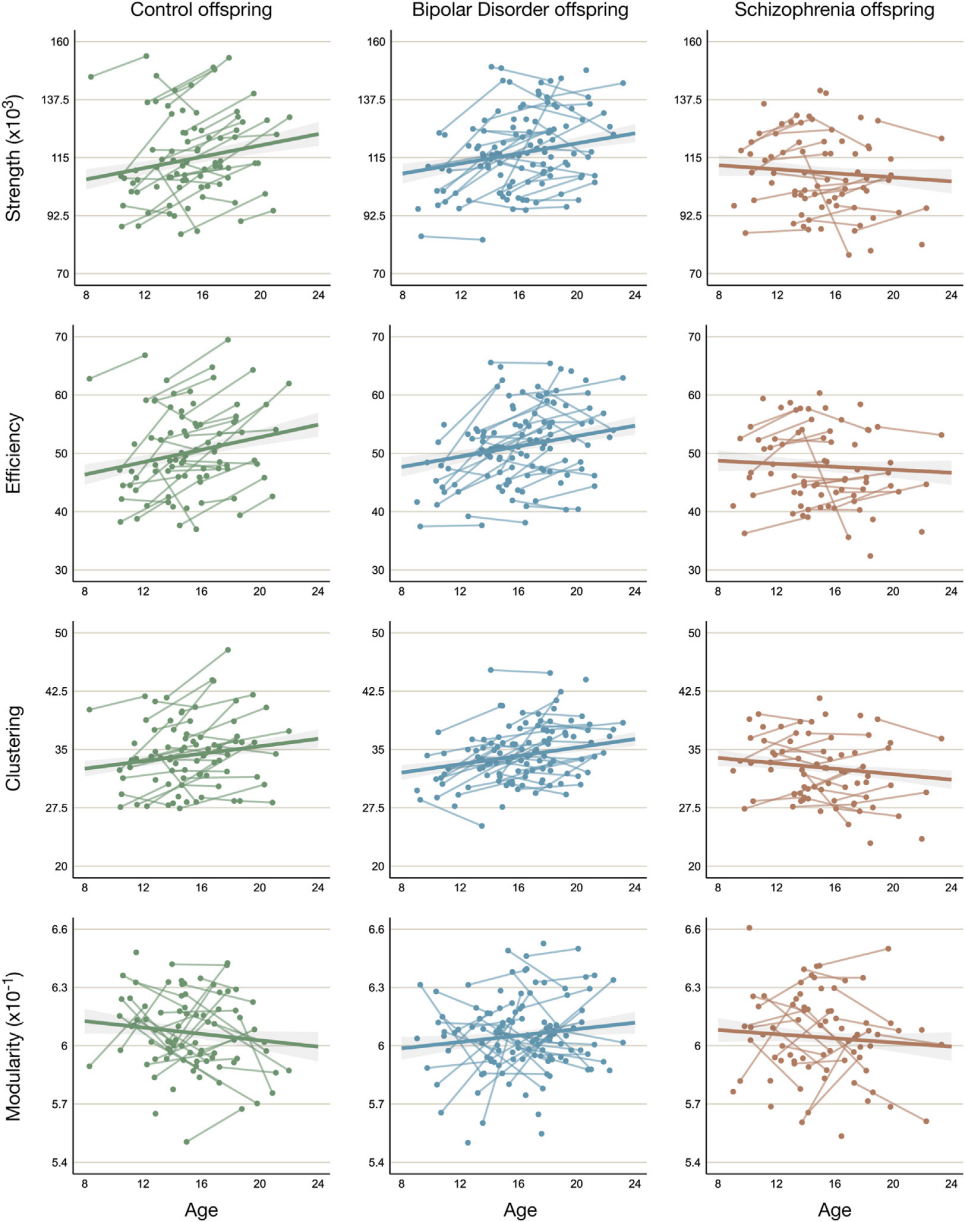
Global network metrics as a function of age per group. Raw data points are presented with the model fit and standard error. The age slopes of offspring of parents with schizophrenia differed significantly from those of offspring of parents with bipolar disorder and offspring of control parents for connectivity strength (*p* = .015 and *p* = .016, respectively) and clustering (*p* = .002 and *p* = .008, respectively) and from that of offspring of control parents for efficiency (*p* = .016).

After correction for multiple comparisons, the linear mixed-model analyses revealed significant age effects in Co in global efficiency (*p* = .010) but not in connectivity strength (*p* = .018), clustering (*p* = .040), or modularity (*p* = .306). With respect to group differences, connectivity strength and clustering followed a significantly different trajectory with age in SZo compared with Co and BDo. Both decreased with age in SZo whereas they increased with age in Co (*p* = .016 and *p* = .008, respectively) and BDo (*p* = .015 and *p* = .002, respectively). SZo differed significantly from Co (*p* = .016) but not from BDo (*p* = .022) in the trajectory of global efficiency. SZo did not differ significantly from Co (*p* = .788) or BDo (*p* = .156) in modularity. Additionally, trajectories of global connectome metrics in BDo did not differ significantly from those in Co (all *p*s ≥ .089) (Table 2). Figure 2 shows the trajectories of each NOS-weighted network metric per group as a function of age. The statistics for the other variables added to the main model (i.e., group, sex, and scanner) are provided in Table S10. Figure S2 illustrates the distribution of sex across the 4 global network metrics.

**Table 2:** Main Effect of Age (Representing the Effect of Age on Network Metric in Co) and Group × Age Interaction Effects for NOS-Weighted Global Network Metrics

Network Metric^a^	Age and Group × Age			
	β	Standard Error	*t*	*p*
Connectivity Strength				
(Intercept)^b^	108,743.85	2545.05	*t*_156.42_ = 42.73	<.001
Age in Co^b^	1100.02	462.44	*t*_181.95_ = 2.38	.018
Age-slope BDo vs. Co^b^	−124.01	538.81	*t*_167.97_ = −0.23	.818
Age-slope SZo vs. Co^b^	−1488.86	613.15	*t*_179.12_ = −2.43	.016^c^
Age-slope SZo vs. BDo^d^	−1364.85	556.39	*t*_198.35_ = −2.45	.015^c^
Global Efficiency				
(Intercept)^b^	47.6	1.12	*t*_156.06_ = 42.60	<.001
Age in Co^b^	0.53	0.20	*t*_183.05_ = 2.61	.010^c^
Age-slope BDo vs. Co^b^	−0.09	0.24	*t*_169.14_ = −0.39	.699
Age-slope SZo vs. Co^b^	−0.66	0.27	*t*_180.35_ = −2.44	.016^c^
Age-slope SZo vs. BDo^d^	−0.57	0.25	*t*_199.69_ = −2.31	.022
Clustering				
(Intercept)^b^	32.59	0.65	*t*_152.93_ = 49.92	<.001
Age in Co^b^	0.24	0.12	*t*_175.86_ = 2.07	.040
Age-slope BDo vs. Co^b^	0.03	0.13	*t*_161.83_ = 0.23	.821
Age-slope SZo vs. Co^b^	−0.41	0.15	*t*_172.53_ = −2.70	.008^c^
Age-slope SZo vs. BDo^d^	−0.44	0.14	*t*_191.30_ = −3.19	.002^c^
Modularity (β and Standard Error × 10^−4^)				
(Intercept)^b^	6073.60	31.39	*t*_179.17_ = 193.51	<.001
Age in Co^b^	−7.71	7.51	*t*_246.99_ = −1.03	.306
Age-slope BDo vs. Co^b^	15.46	9.05	*t*_236.48_ = 1.71	.089
Age-slope SZo vs. Co^b^	2.69	9.99	*t*_245.91_ = 0.27	.788
Age-slope SZo vs. BDo^d^	−12.77	8.98	*t*_255.77_ = −1.42	.156
Rich Club Connectivity				
(Intercept)^b^	13,691.40	672.49	*t*_153.36_ = 20.36	<.001
Age in Co^b^	184.20	111.94	*t*_166.19_ = 1.65	.102
Age-slope BDo vs. Co^b^	−51.43	130.08	*t*_151.58_ = −0.40	.693
Age-slope SZo vs. Co^b^	−265.90	148.15	*t*_161.84_ = −1.79	.074
Age-slope SZo vs. BDo^d^	−214.47	135.96	*t*_175.77_ = −1.58	.116
Feeder Connectivity				
(Intercept)^b^	25,635.62	825.63	*t*_148.12_ = 31.05	<.001
Age in Co^b^	249.84	168.01	*t*_197.42_ = 1.49	.139
Age-slope BDo vs. Co^b^	−91.9	198.31	*t*_185.99_ = −0.46	.644
Age-slope SZo vs. Co^b^	−350.57	222.93	*t*_196.87_ = −1.57	.117
Age-slope SZo vs. BDo^d^	−258.67	201.70	*t*_218.22_ = −1.28	.201
Local Connectivity				
(Intercept)^b^	69,417.82	1533.98	*t*_152.85_ = 45.25	<.001
Age in Co^b^	645.17	279.74	*t*_182.87_ = 2.31	.022
Age-slope BDo vs. Co^b^	4.95	325.71	*t*_168.69_ = 0.02	.988
Age-slope SZo vs. Co^b^	−859.20	370.99	*t*_180.05_ = −2.32	.022
Age-slope SZo vs. BDo^d^	−864.15	336.06	*t*_199.83_ = −2.57	.011^c^

BDo, offspring of parents with bipolar disorder; Co, offspring of control parents; NOS, number of tractography streamlines; SZo, offspring of parents with schizophrenia.

a Linear mixed-model analyses were run for each network metric with group, age, group × age, sex, and scanner as fixed effects and participant and family as random effects.

b Analyses run with Co as reference group.

c Significant after partial Bonferroni correction (*p* < .0167).

d Analyses run with BDo as reference group.

#### RC Organization

**Figure 3:**
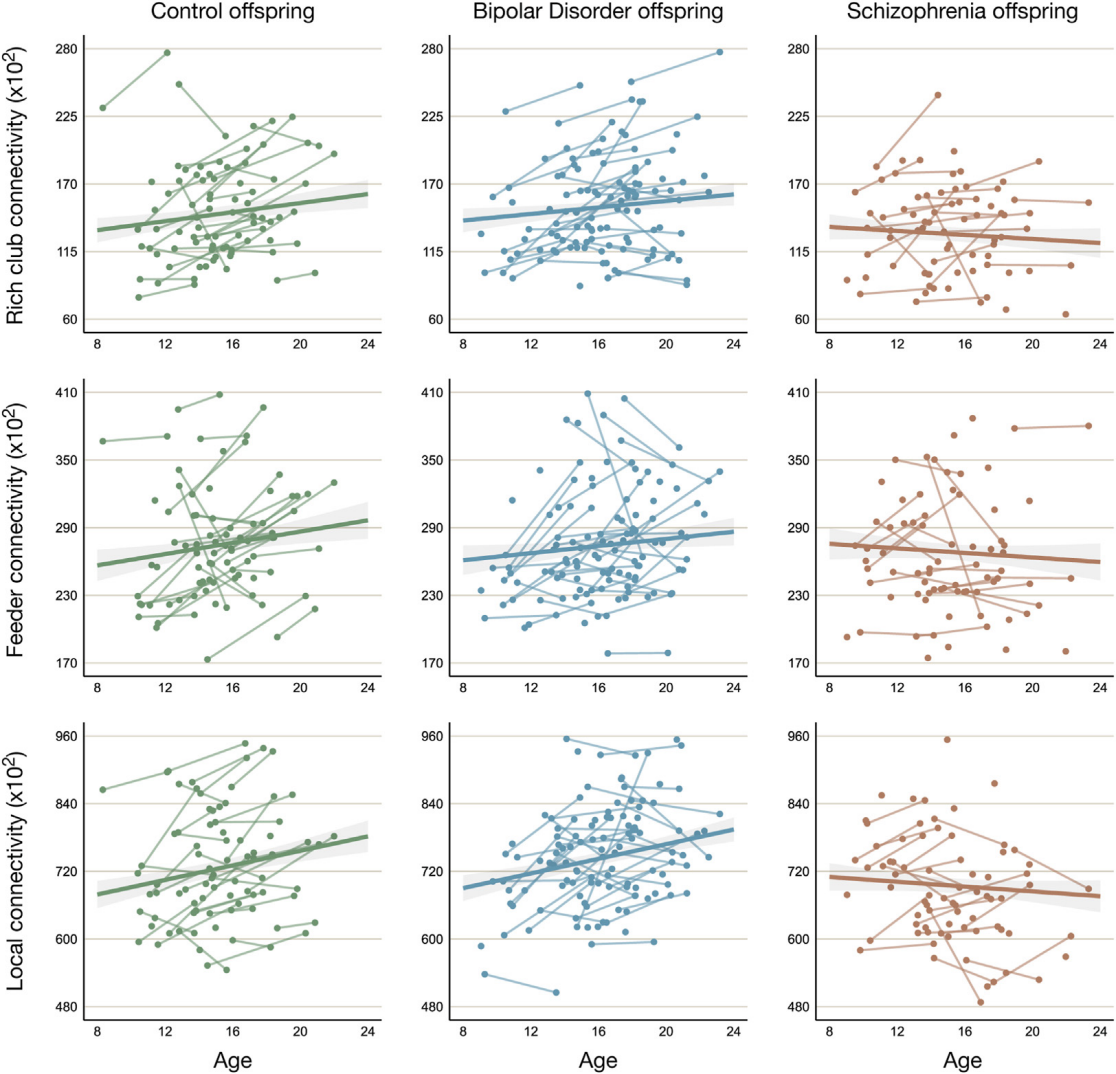
Rich club, feeder, and local connectivity as a function of age per group. Raw data points are presented with the model fit and standard error. The age slope of offspring of parents with schizophrenia differed significantly from that of offspring of parents with bipolar disorder for local connectivity (*p* = .011).

The presence of RC organization was confirmed in the current cohort (Figure S3). Comparison of the 3 group-averaged networks revealed that the hubs were highly similar in the 3 groups (Tables S11 and S12). No significant age effects were found in Co in local connectivity (*p* = .022), RC connectivity (*p* = .102), or feeder connectivity (*p* = .139). Local connectivity decreased with age in SZo whereas it increased in Co (pairwise: *p* = .022) and BDo (pairwise: *p* = .011), reaching statistical significance in comparison with the latter. RC and feeder connectivity age effects did not differ between SZo and Co (*p* = .074 and *p* = .117, respectively) or BDo (*p* = .116 and *p* = .201, respectively). No significant differences were found in age effects in RC, feeder, or local connectivity between BDo and Co (all *p*s ≥ .644) (Table 2). Figure 3 shows the trajectories of the NOS-weighted RC, feeder, and local connectivity per group as a function of age. Repeating the analyses with RC regions selected separately for each group based on their group-averaged network yielded similar results (Tables S13 and S14). Figure S4 illustrates the distribution of sex across RC, feeder, and local connectivity.

### Sensitivity Analyses

Adding IQ, psychotropic medication use (yes/no), or presence of any lifetime psychiatric diagnosis (yes/no) to the main analyses as fixed effects yielded largely similar findings. Group differences in age trajectories of clustering remained significant. While *p* values and betas of the age slope group comparisons of connectivity strength and global efficiency changed only marginally after adding IQ or psychotropic medication use as a covariate, these differences became nonsignificant. Adding presence of any lifetime psychiatric diagnosis as a covariate did not change the results (Tables S15–S17).

## Discussion

In this prospective cross-disorder study, we investigated the development of the structural connectome in child and adolescent offspring at increased familial risk of severe mental illness. We found that SZo had differential trajectories of connectivity strength and clustering compared with both Co and BDo, of global efficiency compared with Co, and of local connectivity compared with BDo. Whereas the trajectories increased in BDo and Co as they grew older, they decreased with increasing age in SZo. The pattern of results remained largely similar after taking IQ, presence of psychopathology, and medication use in offspring into account. These findings suggest that familial high risk of schizophrenia is related to deviations in trajectories of several global connectome characteristics and local (nonhub-to-nonhub) connectivity strength, further increasing our understanding of the neurodevelopmental mechanisms behind familial risk of bipolar disorder and schizophrenia. Moreover, it can inform genetic research on the origins of schizophrenia.

Case-control studies that have investigated the structural connectome in individuals with schizophrenia have mostly shown lower overall connectivity strength (25,29,30,33), global efficiency (30, 31, 32, 33,61,62), and clustering (29,30,33) [but see (24,25,27,29)], with the latter also being lower in siblings (33). Our findings of child and adolescent SZo showing a subtle but significant decline in these structural connectome properties with increasing age suggest that the deviations found after illness onset are possibly partially explained by familial risk of the disorder. The decrease persisted after accounting for the presence of a psychiatric diagnosis, suggesting that disorder-related factors do not play a major role in explaining structural connectome deviations. This hypothesis should be tested in future research by following SZo beyond the mean age of illness onset and examining psychosis onset in the SZo.

In individuals with bipolar disorder, most cross-sectional studies have found unaltered connectivity strength (17,22,23,29), global efficiency (21,23,29,63), and clustering (21,23,29,63), with the latter two also being unaltered in offspring and other relatives (15,35,63). Longitudinally, other white matter measures also generally have not shown significant differences between relatives of individuals with bipolar disorder and control individuals (40,41) [but see (15, 16, 17,36)]. Our study findings are consistent with most studies because no deviating trajectories were found in any of the structural network metrics in BDo compared with Co. In fact, BDo differed significantly from SZo as well, suggesting that familial high risk of bipolar disorder and schizophrenia have differential effects on child and adolescent structural connectome development. This supports findings from another longitudinal cohort study that found different trajectories in cortical morphometric measures between these 2 high-familial-risk offspring groups (64,65).

With respect to RC connectivity, studies have consistently found reduced anatomical RC (hub-to-hub) connectivity in schizophrenia (24, 25, 26, 27,30). Correspondingly, siblings of individuals with schizophrenia exhibit RC disruptions (33). Again, in individuals with bipolar disorder, findings have been more varied, with studies reporting unaltered (17, 18, 19), decreased (20), or increased (21, 22, 23) RC connectivity, and previous studies in BDo also presented no significant differences in RC connectivity (15,34,35) compared with Co. Our cross-sectional findings at time point 1 in child and adolescent offspring are consistent with these patterns, demonstrating suggestive evidence for lower RC connectivity only in SZo (34). Our analyses revealed a nonsignificant effect of familial risk of schizophrenia on the age trajectory of RC connectivity but in the same direction as cross-sectional findings and previous literature. Moreover, local (nonhub-to-nonhub) connectivity decreased significantly with age in SZo but increased in BDo. Given the fact that overall connectivity strength followed this same pattern and that local connectivity still accounts for a large portion of the individual brain networks (the connections between 94 of the total 114 regions), this finding may be driven by a global effect in decreasing connectivity.

The global connectome metrics investigated in this study were strongly intercorrelated, as reported in previous studies (55, 56, 57). We found significant effects in SZo compared with Co and BDo, all in the same direction, in NOS-weighted connectivity strength, global efficiency, and clustering. Because connectivity strength is calculated as the sum of all weighted network connections, a network with higher connectivity strength is likely to contain a higher number of edges. Consequently, such a network has an increased chance of forming clusters by virtue of including more connections in the first place. Similarly, because global efficiency is based on the average shortest path length between all pairs of brain regions, a more interconnected network facilitates fewer steps being needed for one node to be linked to another. The high correlations between these global metrics suggest that the differences in age trajectories found in our analyses may be driven by shared underlying organizational effects. To investigate regional or subnetwork connectivity effects related to familial risk of schizophrenia, these connectome metrics should be assessed at a nodal level, or methods such as network-based statistics may be used (66,67).

It is important to note that most of the offspring in this study have not yet reached the typical age of onset at which the intergenerational homotypic continuity of psychopathology from parent to offspring can be observed (11,12,68). While (sub)clinical symptoms in the psychotic and several other domains are present in both high-familial-risk groups, the number of offspring diagnosed with a bipolar or psychotic disorder in this cohort is very low (69). Because more of them are likely to develop psychosis or bipolar disorder (4, 5, 6), following these offspring further into adulthood is imperative because it will allow us to determine how connectome development pertains to risk of or resilience against later severe mood or psychotic disorder.

Several methodological constraints should be acknowledged when interpreting the findings of this study. First, structural brain networks were obtained using DWI, a technique built on the assumption that the measured water diffusion serves as an indirect marker of axonal orientation (70). As a result, the method suffers from inherent limitations with respect to complex fiber reconstruction (71,72). For connectome mapping in particular, this issue may lead to an underestimation of dysconnectivity effects both within and across offspring groups (73). Second, the individual brain networks consisted exclusively of cortical regions. Although connections between cortical and subcortical regions (e.g., limbic and basal ganglia systems) have been shown to be implicated in bipolar disorder (16) and schizophrenia (74, 75, 76), a recent connectome study in individuals with affective and psychotic disorders that employed methodology similar to the current study demonstrated that adding subcortical regions to the networks yielded findings highly comparable to the cortico-cortical analyses (29). Third, despite the uniqueness of longitudinal assessment of a part of the high-familial-risk and control samples, the modest sample size is a limitation, which may pose a problem in relation to statistical power specifically when considering the heterogeneity of structural connectome data. The number of streamlines derived from diffusion tractography is a notoriously noisy measure of structural connectivity (77). Nevertheless, several studies comparing diffusion tractography and tract tracing have shown streamline count to be a reasonable estimate of connectivity strength (73,78,79). Our sample size also did not enable us to examine sex-related effects because it does not provide sufficient statistical power for a 3-way interaction of sex, age, and group. Because sex was significant as a covariate in our main models, and sex differences in brain development and prevalence of psychiatric disorders have been reported (80, 81, 82, 83, 84), future studies should investigate this in larger samples. Fourth, in-scanner head motion has been shown to systematically bias estimates of DWI-derived structural connectivity in pediatric neuroimaging (85). However, we comprehensively preprocessed our images using advanced retrospective motion correction tools (86, 87, 88, 89, 90), performed rigorous visual quality control, and added movement parameters to our analyses, which did not change our findings. Consequently, we are confident that our findings are not explained by head movement. Fifth, 2 scanners were used for data acquisition. Inevitably, longitudinal studies are especially susceptible to methodological inconsistencies owing to the increased likelihood of practical developments taking place throughout the years, including scanner upgrades, which may introduce bias and reliability issues (91, 92, 93). Therefore, scanner was added as a covariate in the analyses to correct for its effect on the variance. Sixth, data from 2 time points may not provide sufficient information to reliably detect possible nonlinear trends. Additional repeated assessments are needed to capture developmental trajectories in more detail.

### Conclusions

In conclusion, the current study provides evidence for deviant connectome development in offspring at familial risk of schizophrenia during childhood and adolescence as SZo exhibit subtle decreasing age-dependent trajectories of several global structural connectome measures compared with increases found in both Co and BDo. To understand how the deviations in neurodevelopment pertain to risk of mental illness in the offspring, follow up of prospective offspring studies beyond the typical age of illness onset is warranted. Identifying the neurodevelopmental mechanisms that underlie risk of or resilience against psychopathology can open up new avenues for research into preventive treatments for mood and psychotic disorders to reduce risk and/or strengthen resilience.

## Supplementary Material

Key Resources Table

Supplemental Methods and Results
