# Supplementary material for: Age Trajectories of the Structural Connectome in Child and Adolescent Offspring of Individuals With Bipolar Disorder or Schizophrenia
Source: Biol Psychiatry Glob Open Sci. 2024 May 28;4(5):100336. doi: 10.1016/j.bpsgos.2024.100336 (PMC11260845; doi:10.1016/j.bpsgos.2024.100336)
Supplement: Supplemental Methods and Results [file mmc2.pdf]

SUPPLEMENTARY INFORMATION

Age Trajectories of the Structural Connectome in Child and Adolescent Offspring of Individuals with Bipolar Disorder or Schizophrenia

Poortman *et al.*

Supplemental Methods

Participants

Comparing excluded participants (those without available MRI data) with included participants (those with available MRI data on at least one time point) on their demographic and clinical characteristics at inclusion (time point 1) did not reveal any significant differences for age (p = 0.060), group (p = 0.741), sex (p = 0.872), psychotropic medication use (p = 0.592) or presence of any psychiatric diagnosis (p = 0.707). Considering all individual data points, the proportion of total scan exclusions did not differ significantly between the three groups (p = 0.290) (Supplemental Table S1). 33 BDo, 28 SZo and 16 Co had one scan (time point 1: 10 BDo, 12 SZo and 7 Co; time point 2: 23 BDo, 16 SZo and 9 Co), and 41 BDo, 23 SZo and 33 Co had two scans (of the total 74 BDo, 51 SZo and 49 Co) (Supplemental Table S3). There was no significant difference between groups in those with one scan versus those with two scans (p = 0.082). With respect to the number of offspring scanned with each scanner (Achieva or Ingenia CX), a significant group difference was found at time point 1 (p = 0.014), with pairwise comparisons showing a significant difference between BDo and Co (p = 0.004). No significant group differences were found at time point 2 (p = 0.948), nor between offspring who switched scanners and those who did not (p = 0.062) (Supplemental Table S4). Lastly, considering the

entire sample ($N = 286$), to determine whether there are group differences in how many times each of the two scanners was used, and whether the effect of age on which scanner was used differs between groups, we ran a generalized linear model as follows: 'Scanner ~ Group + Age + Group x Age', which showed that there are no significant (age-related) group differences in which of the two scanners was used (all p's > 0.117) (Supplemental Table S5).

Recruitment process and exclusion criteria

Offspring at high familial risk were recruited via adult psychiatrists and child-and-adolescent psychiatrists in the Netherlands, advertisements or hear-say. Control families were recruited via advertisements on schools and leisure clubs or hear-say. Exclusion criteria for all participants were severe physical illness, neurological problems (e.g., open or closed head injury, epilepsy) or an IQ below 70 and, for controls only, a first-degree relative with a severe mood or psychotic disorder.

Instruments

At both time points, participants were psychiatrically evaluated using the Schedule for Affective Disorders and Schizophrenia for School-Age Children-Present and Lifetime Version (1) by interviewing participants and their parents separately. The summary information of this face-to-face semi-structured interview was used to establish the presence and severity of symptoms and current and lifetime DSM-IV axis I diagnoses. IQ was estimated using four subtests (block design, picture completion, information and vocabulary) of the Wechsler Intelligence Scale for Children-III (≤16 years) (2) or the Wechsler Adult Intelligence Scale-III (>16 years) (3).

MRI acquisition

T1-weighted imaging. Three-dimensional T1-weighted sagittal spoiled-gradient fast-field echo scans of the whole brain were acquired for tissue classification and cortical parcellation, with the following

parameters per scanner: *Philips 3 Tesla Achieva.* 200 contiguous slices; 0.75 x 0.75 x 0.80 mm^3 voxel size, TE = 4.6 ms, TR = 10.029 ms, flip angle = 8°, field of view (FOV) = 240 x 240 x 160 mm^3, eight-channel head coil, acquisition time = 10 minutes. *Philips 3 Tesla Ingenia CX.* 200 contiguous slices; 0.75 x 0.75 x 0.80 mm^3 voxel size, TE = 4.6 ms, TR = 10.021 ms, flip angle = 8°, FOV = 240 x 240 x 160 mm^3, eight-channel head coil, acquisition time = 10 minutes.

Diffusion-weighted imaging (DWI). Two-dimensional DWI scans with reversed phase-encoding blips were used for the reconstruction of white matter pathways, using the following parameters per scanner: *Philips 3 Tesla Achieva*: 75 non-angulated slices, 1.875 x 1.875 x 2.00 mm^3 voxel size, TE = 68 ms, TR = 6991.08/7011.21 ms, EPI factor = 33, flip angle = 90°, FOV = 240 x 240 x 150 mm^3, 30 b=1000 s/mm^2 and 5 b=0 s/mm^2 (anterior-posterior), 30 b=1000 s/mm^2 and 5 b=0 s/mm^2 (posterior-anterior), eight-channel head coil, acquisition time = 11 minutes 25/27 seconds. *Philips 3 Tesla Ingenia CX.* 75 non-angulated slices, 1.875 x 1.875 x 2.00 mm^3 voxel size, TE = 69 ms, TR = 7045.89/7109.81 ms, EPI factor = 33, flip angle = 90°, FOV = 240 x 240 x 150 mm^3, 30 b=1000 s/mm^2 and 5 b=0 s/mm^2 (anterior-posterior), 30 b=1000 s/mm^2 and 5 b=0 s/mm^2 (posterior-anterior), eight-channel head coil, acquisition time = 11 minutes 30/36 seconds.

MRI preprocessing

All imaging data were coded to ensure blinding for participant identification and diagnoses during image processing. Imaging data were (pre)processed and analysed on SURFsara's Snellius compute cluster. Before migration to the compute cluster, T1-weighted images were defaced using mri_deface version 1.22 (4). Visual quality control was performed by S.P. to determine whether defacing was performed successfully (i.e., the participant's face was made unrecognisable with the brain left intact).

FreeSurfer (v7.1.1) (5) and the FMRIB Software Library (FSL, v6.0.6) (6) were used to process and segment the T1-weighted image and to perform preprocessing on the DWI images. T1-weighted image processing included automated Talairach transformation, intensity normalisation, removal of

non-brain tissue, segmentation of subcortical white matter and grey matter, tessellation of the grey/white matter boundary, and automated topology correction (7–10). Visual quality control was done according to the ENIGMA procedures (http://enigma.ini.usc.edu/). DWI data were corrected for susceptibility-induced distortions (11), eddy current distortions and motion artefacts (12), outliers (13), intra-volume movement (14) and susceptibility-by-movement (15). The b-vectors were updated to adjust for DWI corrections (16). Visual quality control of the processed DWI data was performed by S.P. and M.B., where each researcher thoroughly inspected each volume of half of the scans, checking residual motion, scanner and metal-induced artefacts, and brain coverage. In case of uncertainty about whether a scan should be excluded or not, the other researcher was included in the process and a decision was reached through consensus. Visual quality control was always done blind to the group the participant was in. After our visual data quality control, we re-evaluated the scan quality of the scans where participants showed a steep change in network metrics. These scans were all of acceptable quality and the steep changes could not be explained by changes in scan quality (e.g., having one borderline scan and one of really good quality). We also did not see group differences in how often participants show a steep change (defined as two standard deviations away from the mean) in connectivity strength (Co: n = 1; BDo: n = 1; SZo: n =1). Therefore, we did not see a reason to exclude these data.

The Connectivity Analysis TOolbox (CATO) (v3.2.1) (17) was used to reconstruct structural connectivity from the processed DWI data. A reference image was computed based on the corrected b=0 volumes. Next, the registration matrix between the DWI reference image and the T1 image was created using bbregister (18). The FreeSurfer segmentation files were registered to the DWI reference image for anatomical alignment. Diffusion directions were determined in each voxel using the informed RESTORE algorithm to reduce the impact of physiological noise artefacts on the DTI modelling (19,20). Deterministic tractography was performed to reconstruct the white matter pathways, using an extended version of the Fiber Assignment by Continuous Tracking algorithm (21).

While advanced reconstruction methods such as those based on crossing-fiber models and probabilistic tractography allow for more complete reconstructions of complex fibre pathways and high sensitivity (22), deterministic tractography was chosen to obtain an adequate false negative to false positive fibre reconstruction ratio, which has been shown to have a substantial impact on connectome analyses (23–25). Fibre reconstruction was initiated from eight seeds in each cerebral white matter voxel. For each seed, a tractography streamline was propagated in the mean diffusion direction from voxel to voxel. Fibre tracking was terminated when (1) FA<0.1, (2) a>45° turn was made, (3) a stop region was entered, (4) a forbidden region was about to be entered, (5) the current or previously visited voxel was about to be revisited or (6) the brain mask was exited.

Structural connectome topology

A selection of global characteristic graph metrics was computed to investigate possible differences in the development of overall connectome topology between the three groups (26) (Figure 1E). The connectivity strength of a brain network was quantified as the sum of the weights of all connections in that network. Global efficiency, representing the network's overall communication capacity, was computed as the harmonic mean of the shortest path length (i.e., fewest number of edges that must be traversed for one node to connect to another) between all pairs of nodes in the network. As a measure of segregation, the clustering coefficient was calculated as the average likelihood that a node's neighbours are also connected among themselves. The modularity represents the degree to which a network can be broken down into community structures (i.e., cliques of nodes with the highest possible number of within-group connections and the lowest possible number of between-groups connections) (27).

Demographic and clinical characteristics

Group differences in demographic and clinical characteristics were evaluated at both time points using Fisher's exact test for categorical variables (i.e., clinical diagnosis, psychotropic medication use and sex) and analyses of variance for continuous variables (i.e., age, IQ and scan interval) followed by Tukey's test in case of significant group effects.

Confounding variables

Several potentially confounding variables are added to our (sensitivity) analyses. Sex was included because there are known differences between boys and girls in age-trajectories of structural brain development (28–31). IQ, medication use and psychopathology are confounders of the familial risk of mental illness: IQ is known to be lower, and medication use and psychopathology are known to be higher in the high-familial-risk groups (32–34). Therefore, we applied sensitivity analyses with these variables, as it allows us to examine whether they explain the group differences, i.e., it enables us to compare group differences before and after adding the covariate.

Statistical assumptions

Normality of the model residuals was visually inspected using histograms and Q-Q plots. Additionally, model residuals were tested for skewness, kurtosis and normality using the D'Agostino, Anscombe-Glynn test and Shapiro-Wilk tests, respectively. Based on these plots and statistics, we concluded that the model residuals are sufficiently normally distributed.

Time point 1 connectome analyses

Analyses to compare the graph theoretical metrics between groups at time point 1, as was done previously in a subgroup of the current sample (35), were repeated on the metrics obtained from the NOS-weighted networks of the time point 1 sample. Specifically, a linear mixed model analysis was performed with group, age and sex as fixed effects and family as a random effect. In contrast to our

previous analyses, in which participants were only scanned in one scanner, scanner was now added as a fixed effect. Also, we now selected rich club nodes a priori instead of defining them for each subject individually. The resulting formula was as follows: 'lmer(NetworkMetric ~ Group + Age + Sex + Scanner + (1|FamilyID))'.

Effects of head motion

In addition to comprehensive preprocessing and rigorous visual quality control of the DWI and T1-weighted scans, we investigated potential effects of head motion statistically. For each DWI dataset, we used the average of the root mean square (RMS) volume-to-volume movement values, accounting for the eddy current component, taken from the *my_eddy_output.eddy_restricted_movements_rms* output file (see: https://fsl.fmrib.ox.ac.uk/fsl/fslwiki/eddy/UsersGuide). To see whether there was an effect of age and whether this differed per group, we ran an analysis of variance (ANOVA) as follows: 'AverageRMSmovement ~ Group + Age + Group x Age'. There was no group difference (Group: p = 0.238) and there was a strong effect of age (Age: $p < .001$), but this did not differ significantly between groups (Group x Age: p = 0.457).

For each T1-weighted scan, we calculated the Euler number for each hemisphere using the surface holes given by FreeSurfer's *recon-all*, and then took the sum of the Euler numbers of both hemispheres for a total Euler number per scan. To see whether there was an effect of age and whether this differed per group, we ran an ANOVA as follows: 'Euler ~ Group + Age + Group x Age'. There was no significant group difference (Group: p = 0.691) and there was a strong effect of age (Age: $p <$ .001), but this did not differ significantly between groups (Group x Age: p = 0.857).

Next, we added both the AverageRMSmovement variable and the Euler number to the main analyses' lmer() models as fixed effects to evaluate their impact on each of the seven outcome measures, resulting in the following formula: 'lmer(NetworkMetric ~ Group + Age + Group x Age +

Sex + Scanner + AverageRMSmovement + Euler + (1|FamilyID/SubjectID))'. Adding these two parameters did not change the nature of our findings (Supplemental Table S7).

Rich club organization

Rich club organization was investigated by normalising the weighted rich club coefficient (36) relative to a set of 10.000 comparable random networks (26,37,38). Specifically, a weighted group-averaged network was reconstructed by constructing a binary network of all connections identified in at least 50% of the participants (participants with scans at both time points and therefore two network matrices had their network matrices averaged first). The weight of each connection in the group-averaged network was averaged across subjects. Next, a weighted rich club curve was computed for the empirical group-network and compared to the average rich club curve of a set of 10.000 comparable randomised networks comprising the same connection weights (26,37,38). Then, a normalised rich club curve was calculated as the ratio between the empirical and random rich club curve (35). The presence of rich club organization is indicated where the normalised rich club curve significantly exceeded 1 over a range of degree k.

To confirm consistent rich club membership across groups, a separate group-averaged network was computed for each group as well. The top 20 high-degree nodes of each group's group-averaged network were compared. Additionally, hubs with a degree of 19 or higher of each group's group-averaged brain network were compared.

Supplemental Results

Time point 1 connectome analyses

Repeating analyses on only those scanned at time point 1 largely aligned with our previous findings (36,37), despite now including individuals scanned at two different scanners and selecting rich club

nodes a priori instead of defining them for each subject individually. We find no significant effects between groups on global network metrics (i.e., connectivity strength, clustering coefficient, global efficiency or modularity), local connectivity or feeder connectivity. Now, SZo showed reduced rich club connectivity compared to Co and BDo, but these differences did not reach statistical significance (p = 0.290 and p = 0.052, respectively) (Supplemental Table S6), while Collin et al. (33) reported significantly reduced rich club connectivity in SZo as compared with Co (p = 0.018) and BDo (p = 0.010).

Rich Club Organization

Rich club organization was verified in the current cohort at k=15, k=16 and over range k=19 to k=23 (Supplemental Figure S3). The top 20 high-degree nodes and the nodes with a degree of 19 or higher of each group's group-averaged network were examined to compare rich club membership between groups, revealing it to be highly consistent between groups (Supplemental Table S11 and S12). Across groups (with edges present in at least 50% of all subjects in the respective group, after averaging the network matrices of those with scans at both time points), the top 20 high-degree nodes included parts of the bilateral inferior parietal gyrus, posterior cingulate gyrus, precuneus, rostral middle frontal gyrus and superior parietal gyrus, the left superior frontal gyrus and superior temporal gyrus and the right insula, isthmus of the cingulate gyrus and postcentral gyrus. In the group connectome based on all participants (with edges present in at least 50% of all subjects, after averaging the network matrices of those with scans at both time points), the top 20 high-degree nodes comprised all of these regions and, additionally, subregions of the left lateral orbitofrontal gyrus and the right lateral occipital gyrus and superior temporal gyrus (Supplemental Table S11). Rich club, feeder and local connectivity analyses with hub selection based on each group's averaged brain network are shown in Supplemental Table S13. Running these analyses with hub selection based on the group connectome of all participants yielded highly similar results (Supplemental Table S13).

FA-weighted networks

Running the analyses on network measures computed on FA-weighted networks yielded a significant age effect in Co in connectivity strength (p<0.001) and global efficiency (p=0.001), but not in clustering (p=0.066) and modularity (p=0.302). There were no significant group differences in age effects in the global metrics computed on the FA-weighted networks (p≥0.072, Supplemental Table S8).

Running the analyses on network measures computed on FA-weighted networks showed a significant age effect in Co in rich club, feeder and local connectivity (all p's<0.001). There were no significant group differences in age effects between the three measures (all p's<0.138).

Supplemental Table S1. Exclusion reason per group per time point.

	Time point 1				Time point 2			
	BDo	SZo	Co	Total	BDo	SZo	Co	Total
N original	99	64	62	225	99	64	62	225
Pathology	4	1	0	5	4	1	0	5
Patient	1	0	0	1	1	0	0	1
1x SDR	0	3	0	3	0	3	0	3
IQ below 70	1	0	0	1	1	0	0	1
MRI incompatible[a]	6	5	0	11	3	1	2	6
Missing scan(s)	1	3	2	6	4	3	0	7
Poor scan quality	15	5	15	36	5	4	4	13
Other	20	12	5	37	7	6	7	20
Dropout	0	0	0	0	10	7	7	24
N total excluded	48	29	22	98	35	25	20	80
N after exclusion	51	35	40	126	64	39	42	145

BDo = bipolar disorder offspring, Co = control offspring, SDR = second-degree relative, SZo = schizophrenia offspring.

[a]Reasons include braces, piercings or metal in the body.

Supplemental Table S2. Frequency of families with one, two or three offspring for each group.

Group	Families with one offspring	Families with two offspring	Families with three offspring
BDo	31	17	3
SZo	23	11	2
Co	19	12	2

BDo = bipolar disorder offspring, Co = control offspring, SZo = schizophrenia offspring.
Fisher's Exact Test showed no significant group differences (p = 0.980).

Supplemental Table S3. Overview of the number of single scans per group per time point and the number of participants scanned at both time points.

	One scan		Both scans
	Time point 1	Time point 2	
BDo	10	23	41
SZo	12	16	23
Co	7	9	33
Total	29	48	97

BDo = bipolar disorder offspring, Co = control offspring, SZo = schizophrenia offspring.

Supplemental Table S4. Number of offspring per group scanned with each scanner at each time point.

	Time point 1		Time point 2		Scanner change[a]	No scanner change[a]	
	Achieva	Ingenia CX	Achieva	Ingenia CX		Achieva	Ingenia CX
BDo	49	2	20	44	28 (68%)	11	2
SZo	30	5	12	27	15 (65%)	6	2
Co	30	10	14	28	14 (42%)	10	9
Total	109	17	46	99	57	27	13

BDo = bipolar disorder offspring, Co = control offspring, SZo = schizophrenia offspring.
[a]These numbers concern the 41 BDo, 23 SZo and 33 Co that were scanned at both time points.

Supplemental Table S5. Generalized linear model to examine (age-related) group differences in which of the two scanners was used.

Scanner (Achieva or Ingenia CX)[a]	Group, Age & Group*Age			
	ß	Standard error	*z*	*p*
(Intercept)[b]	-5.184	1.525	-3.399	<0.001
BDo vs Co[b]	-1.044	2.082	-0.502	0.616
SZo vs Co[b]	1.957	1.997	0.980	0.327
SZo vs BDo[c]	3.001	1.915	1.567	0.117
Age in Co[b]	0.329	0.098	3.352	**<0.001**
Age * BDo vs Co[b]	0.023	0.129	0.177	0.860
Age * SZo vs Co[b]	-0.135	0.128	-1.049	0.294
Age * SZo vs BDo[c]	-0.158	0.117	-1.341	0.180

BDo = bipolar disorder offspring, Co = controls, SZo = schizophrenia offspring.
[a]Generalized linear model were run with scanner as outcome variable and group, age and group*age as predictor variables.
[b]Analyses run with control offspring as reference group.
[c]Analyses run with bipolar disorder offspring as reference group.

Supplemental Table S6. Time point 1 connectome analyses for NOS-weighted global network metrics.

Network metric[a]	Pairwise				
	ß	Standard error	*df*	*t*	*p*
Connectivity strength					
(Intercept)[b]	106952.93	3327.62	110.43	32.14	<0.001
BDo vs Co[b]	4478.20	3448.36	91.45	1.30	0.197
SZo vs Co[b]	-298.81	3659.01	90.10	-0.08	0.935
SZo vs BDo[c]	-4777.00	3431.85	95.44	-1.39	0.167
Global efficiency					
(Intercept)[b]	47.00	1.44	109.90	32.61	<0.001
BDo vs Co[b]	1.70	1.49	89.96	1.14	0.257
SZo vs Co[b]	0.30	1.59	88.55	0.02	0.982
SZo vs BDo[c]	-1.67	1.49	94.03	-1.12	0.264
Clustering					
(Intercept)[b]	32.17	0.81	108.95	39.79	<0.001
BDo vs Co[b]	0.29	0.84	88.11	0.34	0.732
SZo vs Co[b]	0.29	0.89	86.68	-0.33	0.734
SZo vs BDo[c]	-0.58	0.83	92.52	-0.70	0.488
Modularity (*ß* and standard error × 10^{-4})					
(Intercept)[b]	6094.53	44.00	85.34	138.51	<0.001
BDo vs Co[b]	-39.70	44.14	61.32	-0.90	0.372
SZo vs Co[b]	-46.97	46.80	62.33	-1.00	0.319
SZo vs BDo[c]	-7.27	44.47	75.74	-0.16	0.871
Rich club connectivity					
(Intercept)[b]	14077.14	836.09	102.16	16.84	<0.001
BDo vs Co[b]	714.47	848.82	81.28	0.84	0.402
SZo vs Co[b]	-957.90	899.82	80.93	-1.06	0.290
SZo vs BDo[c]	-1672.37	849.88	90.07	-1.97	0.052
Feeder connectivity					
(Intercept)[b]	25425.31	1115.71	108.69	22.79	<0.001
BDo vs Co[b]	618.91	1144.91	90.43	0.54	0.590
SZo vs Co[b]	1139.30	1214.16	89.48	0.94	0.351
SZo vs BDo[c]	520.39	1142.08	95.93	0.46	0.650
Local connectivity					
(Intercept)[b]	67534.13	2010.92	110.49	33.58	<0.001
BDo vs Co[b]	3146.89	2100.33	89.12	1.50	0.138
SZo vs Co[b]	-529.63	2229.86	87.43	-0.24	0.813
SZo vs BDo[c]	-3676.52	2087.54	92.35	-1.76	0.082

BDo = bipolar disorder offspring, Co = controls, NOS = number of streamlines, SZo = schizophrenia offspring.
[a]Linear mixed model analyses were run for each network metric with group, age, sex and scanner as fixed effects, and family as random effect.

[b]Analyses run with control offspring as reference group.
[c]Analyses run with bipolar disorder offspring as reference group.

Supplemental Table S7. Main effect of Age (representing the effect of age on network metric in Co) and Group*Age interaction effects for NOS-weighted global network metrics, corrected for head motion during the DWI and T1-weighted scans.

Network metric[a]	Age & Group*Age				
	ß	Standard error	*df*	*t*	*p*
Connectivity strength					
(Intercept)[b]	107644.20	3132.57	218.74	34.36	<0.001
Age in Co[b]	1113.82	468.21	182.21	2.38	0.018
Age-slope BDo vs Co[b]	-82.43	543.14	168.33	-0.15	0.880
Age-slope SZo vs Co[b]	-1447.57	619.39	180.24	-2.34	0.021
Age-slope SZo vs BDo[c]	-1365.15	563.54	199.38	-2.42	**0.016**
Global efficiency					
(Intercept)[b]	47.34	1.38	218.46	34.33	<0.001
Age in Co[b]	0.53	0.21	183.28	2.58	**0.011**
Age-slope BDo vs Co[b]	-0.08	0.24	169.50	-0.34	0.734
Age-slope SZo vs Co[b]	-0.65	0.27	181.46	-2.36	0.019
Age-slope SZo vs BDo[c]	-0.57	0.25	200.70	-2.27	0.024
Clustering					
(Intercept)[b]	32.04	0.80	214.79	40.21	<0.001
Age in Co[b]	0.26	0.12	174.51	2.21	0.029
Age-slope BDo vs Co[b]	0.05	0.13	160.52	0.33	0.739
Age-slope SZo vs Co[b]	-0.41	0.15	171.88	-2.69	**0.008**
Age-slope SZo vs BDo[c]	-0.46	0.14	190.51	-3.27	**0.001**
Modularity (*ß* and standard error × 10^{-4})					
(Intercept)[b]	6018.97	42.18	182.12	142.69	<0.001
Age in Co[b]	-5.13	7.50	214.90	-0.68	0.495
Age-slope BDo vs Co[b]	16.81	8.97	224.73	1.87	0.062
Age-slope SZo vs Co[b]	1.59	9.91	217.23	0.16	0.872
Age-slope SZo vs BDo[c]	-15.22	8.93	239.93	-1.70	0.090
Rich club connectivity					
(Intercept)[b]	13886.82	806.19	210.63	17.23	<0.001
Age in Co[b]	173.23	112.66	165.27	1.54	0.126
Age-slope BDo vs Co[b]	-53.53	130.29	150.49	-0.41	0.682
Age-slope SZo vs Co[b]	-242.14	148.70	161.35	-1.63	0.105
Age-slope SZo vs BDo[c]	-188.62	136.94	174.57	-1.38	0.170
Feeder connectivity					
(Intercept)[b]	24999.69	1052.62	211.01	23.75	<0.001
Age in Co[b]	259.77	169.44	196.59	1.53	0.127
Age-slope BDo vs Co[b]	-72.95	198.97	185.27	-0.37	0.714
Age-slope SZo vs Co[b]	-329.35	244.00	196.54	-1.47	0.143
Age-slope SZo vs BDo[c]	-256.40	203.30	218.05	-1.26	0.209
Local connectivity					
(Intercept)[b]	68797.68	1887.59	216.58	36.45	<0.001
Age in Co[b]	662.28	283.53	183.46	2.34	0.021
Age-slope BDo vs Co[b]	23.68	328.83	169.45	0.07	0.943
Age-slope SZo vs Co[b]	-867.76	375.21	181.58	-2.31	0.022
Age-slope SZo vs BDo[c]	-891.45	340.75	201.38	-2.62	**0.010**

BDo = bipolar disorder offspring, Co = controls, NOS = number of streamlines, SZo = schizophrenia offspring.
[a]Linear mixed model analyses were run for each network metric with group, age, group*age, sex, scanner, average RMS movement and Euler number as fixed effects, and subject and family as random effects.
[b]Analyses run with control offspring as reference group.
[c]Analyses run with bipolar disorder offspring as reference group.

Supplemental Table S8. Main effect of Age (representing the effect of age on network metric in Co) and Group*Age interaction effects for FA-weighted global network metrics.

Network metric[a]	Age & Group*Age				
	ß (× 10^{-4})	Standard error (× 10^{-4})	*df*	*t*	*p*
Connectivity strength					
(Intercept)[b]	4274.41	22.28	148.58	191.82	<0.001
Age in Co[b]	19.37	3.86	170.52	5.02	**<0.001**
Age-slope BDo vs Co[b]	-5.56	4.48	156.45	-1.24	0.216
Age-slope SZo vs Co[b]	-8.32	5.12	166.75	-1.63	0.106
Age-slope SZo vs BDo[c]	-2.76	4.65	185.30	-0.59	0.554
Global efficiency					
(Intercept)[b]	2180.48	16.14	147.44	135.10	<0.001
Age in Co[b]	10.94	3.20	194.02	3.42	**0.001**
Age-slope BDo vs Co[b]	-4.48	3.76	182.32	-1.19	0.235
Age-slope SZo vs Co[b]	-3.81	4.25	192.80	-0.90	0.371
Age-slope SZo vs BDo[c]	0.68	3.8	214.97	0.18	0.860
Clustering					
(Intercept)[b]	1979.04	13.38	155.09	147.86	<0.001
Age in Co[b]	5.28	2.86	210.13	1.85	0.066
Age-slope BDo vs Co[b]	1.20	3.39	201.37	0.35	0.723
Age-slope SZo vs Co[b]	-4.97	3.79	210.42	-1.31	0.191
Age-slope SZo vs BDo[c]	-6.17	3.42	231.25	-1.81	0.072
Modularity					
(Intercept)[b]	4649.53	35.72	137.11	130.18	<0.001
Age in Co[b]	-9.24	8.94	249.75	-1.03	0.302
Age-slope BDo vs Co[b]	6.19	10.71	259.53	0.58	0.564
Age-slope SZo vs Co[b]	-7.77	11.88	249.55	-0.65	0.514
Age-slope SZo vs BDo[c]	-13.96	10.50	259.13	-1.33	0.185
Rich club connectivity					
(Intercept)[b]	4590.09	31.08	142.35	147.68	<0.001
Age in Co[b]	22.42	5.82	180.58	3.85	**<0.001**
Age-slope BDo vs Co[b]	-9.62	6.80	167.16	-1.42	0.159
Age-slope SZo vs Co[b]	-11.51	7.72	178.08	-1.49	0.138
Age-slope SZo vs BDo[c]	-1.89	6.99	199.97	-0.27	0.787
Feeder connectivity					
(Intercept)[b]	4432.44	23.86	148.59	185.74	<0.001
Age in Co[b]	19.14	4.61	193.86	4.15	**<0.001**
Age-slope BDo vs Co[b]	-5.64	5.39	180.26	-1.05	0.296
Age-slope SZo vs Co[b]	-8.76	6.11	191.92	-1.43	0.154
Age-slope SZo vs BDo[c]	-3.11	5.51	213.24	-0.56	0.573
Local connectivity					
(Intercept)[b]	4162.69	22.97	148.60	181.26	<0.001
Age in Co[b]	17.57	4.08	173.32	4.30	**<0.001**
Age-slope BDo vs Co[b]	-3.64	4.75	159.22	-0.76	0.446
Age-slope SZo vs Co[b]	-7.25	5.41	170.03	-1.34	0.182
Age-slope SZo vs BDo[c]	-3.61	4.92	189.05	-0.73	0.464

BDo = bipolar disorder offspring, Co = controls, FA = fractional anisotropy, SZo = schizophrenia offspring.

[a]Linear mixed model analyses were run for each network metric with group, age, group*age, sex and scanner as fixed effects, and subject and family as random effects.

[b]Analyses run with control offspring as reference group.

[c]Analyses run with bipolar disorder offspring as reference group.

Supplemental Table S9. Principal component analysis loadings (correlations between the z-scores of the NOS-weighted variables and the principal components) and results.

	PC1	PC2	PC3	PC4	PC5	PC6	PC7
**Entire dataset (with those with two scans averaged first, n = 174)**							
Loadings (z-scores versus PCs)							
Connectivity strength	0.450	-0.025	0.034	-0.066	-0.210	-0.307	-0.808
Global efficiency	0.443	0.050	0.059	-0.088	-0.264	0.849	<-0.001
Clustering	0.427	-0.154	0.083	-0.044	0.885	0.051	<-0.001
Modularity	-0.028	-0.951	-0.114	0.244	-0.133	0.062	<-0.001
Rich club connectivity	0.320	0.087	-0.910	-0.045	-0.048	-0.129	0.203
Feeder connectivity	0.387	0.179	0.201	0.816	-0.123	-0.180	0.252
Local connectivity	0.408	-0.168	0.327	-0.508	-0.262	-0.359	0.492
PCA results							
Standard deviation	2.2115	1.0343	0.7696	0.5557	0.3164	0.1965	<0.0001
Proportion of variance	0.6986	0.1528	0.0846	0.0441	0.0143	0.0055	<0.0001
Cumulative proportion	0.6986	0.8515	0.9361	0.9802	0.9945	1.0000	1.0000
**Time point 1 only (n = 126)**							
PCA results							
Standard deviation	2.2139	1.0051	0.7690	0.5809	0.3457	0.2006	<0.0001
Proportion of variance	0.7002	0.1443	0.0845	0.0482	0.0171	0.0058	<0.0001
Cumulative proportion	0.7002	0.8445	0.9290	0.9772	0.9943	1.0000	1.0000
**Time point 2 only (n = 145)**							
PCA results							
Standard deviation	2.2192	1.0313	0.7600	0.5451	0.3100	0.2017	<0.0001
Proportion of variance	0.7036	0.1519	0.0825	0.0425	0.0137	0.0058	<0.0001
Cumulative proportion	0.7036	0.8555	0.9380	0.9805	0.9942	1.0000	1.0000

NOS = number of streamlines, PC = principal component, PCA = principal component analysis.

Supplemental Table S10. Effects of group, sex and scanner in the main linear mixed-effects model analyses for NOS-weighted global network metrics.

Network metric[a]	Group, Sex & Scanner				
	ß	Standard error	*df*	*t*	*p*
Connectivity strength					
(Intercept)[b]	108743.85	2545.05	156.42	42.73	<0.001
BDo vs Co[b]	1320.62	2839.00	118.51	0.47	0.643
SZo vs Co[b]	-5828.64	3070.44	121.44	-1.90	0.060
SZo vs BDo[c]	-7149.27	2824.12	126.49	-2.53	**0.013**
Sex	10484.23	1982.44	154.46	5.29	**<0.001**
Scanner	3138.50	1634.75	242.04	1.92	0.056
Global efficiency					
(Intercept)[b]	47.60	1.12	156.06	42.60	<0.001
BDo vs Co[b]	0.61	1.24	117.77	0.49	0.626
SZo vs Co[b]	-2.64	1.35	120.82	-1.96	0.052
SZo vs BDo[c]	-3.25	1.24	125.95	-2.63	**0.010**
Sex	4.53	0.88	155.29	5.18	**<0.001**
Scanner	1.66	0.72	242.54	2.30	0.023
Clustering					
(Intercept)[b]	32.59	0.65	152.93	49.92	<0.001
BDo vs Co[b]	-0.29	0.73	114.83	-0.39	0.694
SZo vs Co[b]	-1.76	0.79	117.54	-2.23	0.028
SZo vs BDo[c]	-1.47	0.73	122.60	-2.03	0.045
Sex	3.05	0.51	149.55	6.02	**<0.001**
Scanner	0.84	0.41	238.40	2.03	0.044
Modularity (*ß* and standard error × 10^{-4})					
(Intercept)[b]	6073.60	31.39	179.17	193.51	<0.001
BDo vs Co[b]	-14.41	32.41	149.32	-0.44	0.657
SZo vs Co[b]	-22.30	35.48	158.21	-0.63	0.531
SZo vs BDo[c]	-7.89	33.05	162.84	-0.24	0.812
Sex	-8.91	27.39	160.28	-0.33	0.746
Scanner	21.86	25.14	261.55	-0.87	0.385
Rich club connectivity					
(Intercept)[b]	13691.40	672.49	153.36	20.36	<0.001
BDo vs Co[b]	401.91	746.75	115.17	0.54	0.591
SZo vs Co[b]	-1754.29	808.31	118.06	-2.17	0.032
SZo vs BDo[c]	-2156.20	744.23	123.67	-2.90	**0.004**
Sex	621.42	556.73	165.30	1.12	0.266
Scanner	1530.08	408.51	226.41	3.75	**<0.001**
Feeder connectivity					
(Intercept)[b]	25635.62	825.63	148.12	31.05	<0.001
BDo vs Co[b]	-244.03	894.04	106.15	-0.27	0.785
SZo vs Co[b]	-746.93	970.09	111.00	-0.77	0.443
SZo vs BDo[c]	-502.90	896.30	117.12	-0.56	0.576
Sex	2958.41	684.04	161.24	4.32	**<0.001**
Scanner	1280.74	580.07	246.38	2.21	0.028
Local connectivity					
(Intercept)[b]	69417.82	1533.98	152.85	45.25	<0.001
BDo vs Co[b]	1168.49	1716.06	114.54	0.68	0.497
SZo vs Co[b]	-3399.12	1855.50	117.37	-1.83	0.070
SZo vs BDo[c]	-4567.61	1705.86	122.37	-2.68	**0.008**
Sex	6852.44	1176.83	148.82	5.82	**<0.001**
Scanner	474.10	985.85	243.26	0.48	0.631

BDo = bipolar disorder offspring, Co = controls, NOS = number of streamlines, SZo = schizophrenia offspring.

[a]Linear mixed model analyses were run for each network metric with group, age, group*age, sex and scanner as fixed effects, and subject and family as random effects.

[b]Analyses run with control offspring as reference group.

[c]Analyses run with bipolar disorder offspring as reference group.

Supplemental Table S11. Top 20 high-degree nodes per group based on each group's average network. Top 20 high-degree nodes in the group-averaged network of all participants are in italic.

BDo, SZo & Co	BDo & SZo	BDo	SZo	Co
**left hemisphere:**	**left hemisphere:**	**right hemisphere:**	**right hemisphere:**	**left hemisphere:**
inferiorparietal_1	insula_1	superiorparietal_2	*lateraloccipital_1*	precentral_4
posteriorcingulate_1	*lateralorbitofrontal_2*			
precuneus_1				**right hemisphere:**
precuneus_2				precuneus_1
rostralmiddlefrontal_3				*superiortemporal_2*
superiorfrontal_1				
superiorfrontal_4				
superiorparietal_3				
superiortemporal_2				
**right hemisphere:**				
inferiorparietal_3				
isthmuscingulate_1				
postcentral_2				
posteriorcingulate_1				
precuneus_2				
rostralmiddlefrontal_2				
superiorparietal_3				
insula_2				

BDo = bipolar disorder offspring, Co = control offspring, SZo = schizophrenia offspring.

Supplemental Table S12. Hubs with degree $k \geq 19$ per group based on each group's average network (BDo: n = 15; SZo: n = 12; Co: n = 16).

BDo, SZo & Co	BDo & SZo	BDo & Co	SZo & Co	BDo	Co
**left hemisphere:**	**right hemisphere:**	**left hemisphere:**	**right hemisphere:**	**left hemisphere:**	**left hemisphere:**
precuneus_1	inferiorparietal_3	posteriorcingulate_1	posteriorcingulate_1	lateralorbitofrontal_2	inferiorparietal_1
precuneus_2	superiorparietal_3				superiorfrontal_1
rostralmiddlefrontal_3		**right hemisphere:**		**right hemisphere:**	superiorparietal_3
superiorfrontal_4		postcentral_2		superiorparietal_2	
superiortemporal_2					**right hemisphere:**
					superioritemporal_2
**right hemisphere:**					
precuneus_2					
insula_2					
isthmuscingulate_1					
rostralmiddlefrontal_2					

BDo = bipolar disorder offspring, Co = control offspring, SZo = schizophrenia offspring.

Supplemental Table S13. Main effect of Age (representing the effect of age on network metric in Co) and Group*Age interaction effects for NOS-weighted rich club, feeder and local connectivity with top 20 high-degree nodes selected separately for each group based on their group-averaged network, or based on the total sample's group-averaged network (same selection for each group).

Network metric[a]	Age & Group*Age				
	ß	Standard error	*df*	*t*	*p*
**Top 20 nodes taken for each group separately based on their group-averaged network**					
Rich club connectivity					
(Intercept)[b]	7674.61	324.9	151.12	23.62	<0.001
Age in Co[b]	40.27	68.37	206.17	0.59	0.557
Age-slope BDo vs Co[b]	-6.02	81.08	196.48	-0.07	0.941
Age-slope SZo vs Co[b]	-148.07	90.76	206.33	-1.63	0.104
Age-slope SZo vs BDo[c]	-142.05	82.09	226.56	-1.73	0.085
Feeder connectivity					
(Intercept)[b]	36078.19	971.85	153.74	37.12	<0.001
Age in Co[b]	445.03	184.19	186.58	2.42	**0.017**
Age-slope BDo vs Co[b]	-88.10	215.92	172.82	-0.41	0.684
Age-slope SZo vs Co[b]	-499.74	244.25	184.58	-2.05	0.042
Age-slope SZo vs BDo[c]	-411.64	221.90	203.85	-1.86	0.065
Local connectivity					
(Intercept)[b]	65054.75	1591.53	153.70	40.88	<0.001
Age in Co[b]	588.75	284.16	180.43	2.07	0.040
Age-slope BDo vs Co[b]	-32.68	330.30	166.46	-0.10	0.921
Age-slope SZo vs Co[b]	-840.97	376.80	177.23	-2.23	0.027
Age-slope SZo vs BDo[c]	-808.29	341.74	196.44	-2.37	0.019
**Top 20 nodes based on the group-averaged network of the total sample**					
Rich club connectivity					
(Intercept)[b]	6865.24	316.57	153.79	21.69	<0.001
Age in Co[b]	36.46	67.58	211.94	0.54	0.590
Age-slope BDo vs Co[b]	-3.45	80.02	202.20	-0.04	0.966
Age-slope SZo vs Co[b]	-159.53	89.68	212.02	-1.78	0.077
Age-slope SZo vs BDo[c]	-156.07	80.73	232.65	-1.93	0.054
Feeder connectivity					
(Intercept)[b]	36321.79	965.59	156.25	37.62	<0.001
Age in Co[b]	464.42	180.38	187.22	2.57	**0.011**
Age-slope BDo vs Co[b]	-149.8	210.88	173.42	-0.71	0.478
Age-slope SZo vs Co[b]	-447.75	239.19	184.96	-1.87	0.063
Age-slope SZo vs BDo[c]	-297.94	217.03	204.14	-1.37	0.171
Local connectivity					
(Intercept)[b]	65546.61	1579.14	153.96	41.51	<0.001
Age in Co[b]	563.99	290.64	183.43	1.94	0.054
Age-slope BDo vs Co[b]	47.25	339.07	169.69	0.14	0.889
Age-slope SZo vs Co[b]	-885.09	385.38	180.82	-2.30	0.023
Age-slope SZo vs BDo[c]	-932.34	349.57	200.48	-2.67	**0.008**

BDo = bipolar disorder offspring, Co = controls, NOS = number of streamlines, SZo = schizophrenia offspring.
[a]Linear mixed model analyses were run for each network metric with group, age, group*age, sex and scanner as fixed effects, and subject and family as random effects.
[b]Analyses run with control offspring as reference group.
[c]Analyses run with bipolar disorder offspring as reference group.

Supplemental Table S14. Main effect of Age (representing the effect of age on network metric in Co) and Group*Age interaction effects for NOS-weighted rich club, feeder and local connectivity with nodes with a degree of 19 or higher selected separately for each group based on their group-averaged network.

Network metric[a]	Age & Group*Age				
	ß	Standard error	*df*	*t*	*p*
Rich club connectivity					
(Intercept)[b]	4674.77	219.21	146.87	21.33	<0.001
Age in Co[b]	-15.5	48.08	224.52	-0.32	0.747
Age-slope BDo vs Co[b]	23.28	57.08	219.40	0.41	0.684
Age-slope SZo vs Co[b]	-30.10	63.81	225.13	-0.47	0.638
Age-slope SZo vs BDo[c]	-53.38	57.33	243.02	-0.93	0.353
Feeder connectivity					
(Intercept)[b]	31777.37	842.21	155.83	37.73	<0.001
Age in Co[b]	369.23	162.23	188.45	2.28	0.024
Age-slope BDo vs Co[b]	-73.65	190.72	175.06	-0.39	0.700
Age-slope SZo vs Co[b]	-461.48	215.18	186.78	-2.14	0.033
Age-slope SZo vs BDo[c]	-387.83	195.65	205.89	-1.98	0.049
Local connectivity					
(Intercept)[b]	72306.22	1806.69	153.94	40.02	<0.001
Age in Co[b]	728.48	317.15	178.46	2.30	0.023
Age-slope BDo vs Co[b]	-74.39	367.73	164.41	-0.20	0.840
Age-slope SZo vs Co[b]	-990.04	420.65	174.90	-2.35	0.020
Age-slope SZo vs BDo[c]	-915.65	381.12	194.24	-2.40	0.017

BDo = bipolar disorder offspring, Co = controls, NOS = number of streamlines, SZo = schizophrenia offspring.
[a]Linear mixed model analyses were run for each network metric with group, age, group*age, sex and scanner as fixed effects, and subject and family as random effects.
[b]Analyses run with control offspring as reference group.
[c]Analyses run with bipolar disorder offspring as reference group.

Supplemental Table S15. Main effect of Age (representing the effect of age on network metric in Co) and Group*Age interaction effects for NOS-weighted global network metrics with IQ added as covariate

Network metric[a]	*ß*	Standard error	*df*	*t*	*p*
Connectivity strength					
(Intercept)[b]	94972.42	6748.16	253.95	14.07	<0.001
Age in Co[b]	1119.47	461.36	183.31	2.43	**0.016**
Age-slope BDo vs Co[b]	-146.69	538.25	169.62	-0.27	0.786
Age-slope SZo vs Co[b]	-1436.42	612.11	181.23	-2.35	0.020
Age-slope SZo vs BDo[c]	-1289.74	556.12	199.68	-2.32	0.021
Sex	9611.70	2006.29	158.62	4.79	**<0.001**
Scanner	3530.17	1633.44	242.99	2.16	0.032
IQ	123.67	56.31	261.17	2.20	0.029
Global efficiency					
(Intercept)[b]	42.03	2.98	252.56	14.11	<0.001
Age in Co[b]	0.54	0.20	184.45	2.66	**0.008**
Age-slope BDo vs Co[b]	-0.10	0.24	170.77	-0.43	0.668
Age-slope SZo vs Co[b]	-0.64	0.27	182.55	-2.37	0.019
Age-slope SZo vs BDo[c]	-0.54	0.25	201.04	-2.19	0.029
Sex	4.17	0.89	160.09	4.70	**<0.001**
Scanner	1.80	0.72	243.26	2.50	**0.013**
IQ	0.05	0.02	260.57	2.02	0.045
Clustering					
(Intercept)[b]	30.49	1.72	255.56	17.70	<0.001
Age in Co[b]	0.24	0.12	176.22	2.09	0.038
Age-slope BDo vs Co[b]	0.03	0.14	162.25	0.20	0.844
Age-slope SZo vs Co[b]	-0.41	0.15	173.59	-2.65	**0.009**
Age-slope SZo vs BDo[c]	-0.43	0.14	191.68	-3.10	**0.002**
Sex	2.92	0.52	153.85	5.66	**<0.001**
Scanner	0.90	0.41	239.41	2.16	0.031
IQ	0.02	0.01	261.83	1.31	0.190
Modularity (*ß* and standard error × 10^{-4})					
(Intercept)[b]	6102.89	95.16	220.92	64.13	<0.001
Age in Co[b]	-7.80	7.53	245.54	-1.04	0.301
Age-slope BDo vs Co[b]	15.55	9.06	234.74	1.72	0.087
Age-slope SZo vs Co[b]	2.46	10.02	244.54	0.25	0.806
Age-slope SZo vs BDo[c]	-13.09	9.05	253.40	-1.45	0.149
Sex	-6.89	28.15	161.98	-0.24	0.807
Scanner	-22.36	25.23	260.69	-0.89	0.376
IQ	-0.26	-0.81	223.45	-0.33	0.745
Rich club connectivity					
(Intercept)[b]	13317.16	1755.45	252.56	7.59	<0.001
Age in Co[b]	184.82	112.26	165.32	1.65	0.102
Age-slope BDo vs Co[b]	-51.96	130.46	150.77	-0.40	0.691
Age-slope SZo vs Co[b]	-264.47	148.73	161.28	-1.78	0.077
Age-slope SZo vs BDo[c]	-212.51	136.66	174.48	-1.55	0.122
Sex	595.77	567.45	169.15	1.05	0.295
Scanner	1539.80	411.47	227.21	3.74	**<0.001**
IQ	3.38	14.57	254.23	0.23	0.817
Feeder connectivity					
(Intercept)[b]	21466.68	2326.95	235.59	9.23	<0.001
Age in Co[b]	265.86	166.59	194.41	1.60	0.112
Age-slope BDo vs Co[b]	-106.87	196.68	183.55	-0.54	0.588
Age-slope SZo vs Co[b]	-335.68	221.27	194.54	-1.52	0.131
Age-slope SZo vs BDo[c]	-228.81	200.85	214.86	-1.14	0.256
Sex	2669.59	699.85	164.41	3.82	**<0.001**
Scanner	1355.72	577.56	243.69	2.35	0.020
IQ	37.69	19.59	246.57	1.92	0.056
Local connectivity					
(Intercept)[b]	59961.61	4045.25	253.13	14.82	<0.001
Age in Co[b]	644.20	279.84	186.31	2.30	0.022
Age-slope BDo vs Co[b]	1.79	326.36	172.40	0.01	0.996
Age-slope SZo vs Co[b]	-822.91	371.36	184.41	-2.22	0.028
Age-slope SZo vs BDo[c]	-824.70	336.41	203.63	-2.45	**0.015**
Sex	6266.87	1179.88	151.96	5.31	**<0.001**
Scanner	787.88	985.02	245.45	0.80	0.425
IQ	84.59	33.74	260.32	2.51	**0.013**

BDo = bipolar disorder offspring, Co = controls, NOS = number of streamlines, SZo = schizophrenia offspring.

[a]Linear mixed model analyses were run for each network metric with group, age, group*age, sex, scanner and IQ as fixed effects, and subject and family as random effects.

[b]Analyses run with control offspring as reference group.

[c]Analyses run with bipolar disorder offspring as reference group.

Supplemental Table S16. Main effect of Age (representing the effect of age on network metric in Co) and Group*Age interaction effects for NOS-weighted global network metrics with psychotropic medication use (yes/no) added as covariate

Network metric[a]	*ß*	Standard error	*df*	*t*	*p*
Connectivity strength					
(Intercept)[b]	108798.14	2545.00	156.21	42.75	<0.001
Age in Co[b]	1120.58	464.14	183.28	2.41	0.017
Age-slope BDo vs Co[b]	-111.29	539.87	167.37	-0.21	0.837
Age-slope SZo vs Co[b]	-1454.53	615.38	179.21	-2.36	0.019
Age-slope SZo vs BDo[c]	-1343.24	557.89	199.08	-2.41	0.017
Sex	10513.85	1981.76	154.07	5.31	**<0.001**
Scanner	3203.21	1637.66	240.45	1.96	0.052
Psychotropic medication use	-2127.09	2552.95	239.97	-0.83	0.406
Global efficiency					
(Intercept)[b]	47.63	1.12	156.06	42.62	<0.001
Age in Co[b]	0.54	0.21	184.72	2.64	**0.009**
Age-slope BDo vs Co[b]	-0.08	0.24	168.91	-0.35	0.725
Age-slope SZo vs Co[b]	-0.64	0.27	180.81	-2.36	0.019
Age-slope SZo vs BDo[c]	-0.56	0.25	200.83	-2.26	0.025
Sex	4.55	0.87	154.55	5.21	**<0.001**
Scanner	1.69	0.72	241.19	2.34	0.020
Psychotropic medication use	-1.08	1.13	240.55	-0.96	0.339
Clustering					
(Intercept)[b]	32.60	0.65	152.88	49.88	<0.001
Age in Co[b]	0.24	0.12	177.05	2.10	0.037
Age-slope BDo vs Co[b]	0.03	0.13	161.05	0.24	0.812
Age-slope SZo vs Co[b]	-0.41	0.15	172.40	-2.66	**0.009**
Age-slope SZo vs BDo[c]	-0.44	0.14	191.82	-3.15	**0.002**
Sex	3.06	0.51	149.18	6.02	**<0.001**
Scanner	0.84	0.41	236.67	2.05	0.042
Psychotropic medication use	-0.37	0.64	236.45	-0.58	0.563
Modularity (*ß* and standard error × 10^{-4})					
(Intercept)[b]	6073.37	31.45	119.57	193.08	<0.001
Age in Co[b]	-7.96	7.53	216.69	-1.06	0.291
Age-slope BDo vs Co[b]	15.17	9.07	226.16	1.67	0.096
Age-slope SZo vs Co[b]	2.25	10.03	221.78	0.22	0.823
Age-slope SZo vs BDo[c]	-12.92	8.99	242.88	-1.44	0.152
Sex	-9.64	27.42	160.64	-0.35	0.726
Scanner	-23.24	25.23	237.39	-0.92	0.358
Psychotropic medication use	29.75	39.52	253.96	0.75	0.452
Rich club connectivity					
(Intercept)[b]	13695.59	673.85	153.02	20.32	<0.001
Age in Co[b]	185.85	112.54	167.56	1.65	0.101
Age-slope BDo vs Co[b]	-50.87	130.40	150.98	-0.39	0.697
Age-slope SZo vs Co[b]	-263.68	148.84	161.75	-1.77	0.078
Age-slope SZo vs BDo[c]	-212.81	136.53	176.45	-1.56	0.121
Sex	623.94	557.59	164.82	1.12	0.265
Scanner	1532.20	409.46	225.06	3.74	**<0.001**
Psychotropic medication use	-136.00	659.92	239.74	-0.21	0.837
Feeder connectivity					
(Intercept)[b]	25644.93	826.25	147.67	31.04	<0.001
Age in Co[b]	254.23	168.62	198.43	1.51	0.133
Age-slope BDo vs Co[b]	-87.55	198.82	185.12	-0.44	0.660
Age-slope SZo vs Co[b]	-340.30	223.87	196.89	-1.52	0.130
Age-slope SZo vs BDo[c]	-252.75	202.28	218.74	-1.25	0.213
Sex	2969.28	684.27	160.38	4.34	**<0.001**
Scanner	1301.09	581.66	244.77	2.24	0.026
Psychotropic medication use	-543.84	916.44	253.21	-0.59	0.553
Local connectivity					
(Intercept)[b]	69457.13	1532.80	152.76	45.31	<0.001
Age in Co[b]	660.22	280.55	184.17	2.35	0.020
Age-slope BDo vs Co[b]	12.12	326.18	168.05	0.04	0.970
Age-slope SZo vs Co[b]	-838.94	372.07	180.15	-2.25	0.025
Age-slope SZo vs BDo[c]	-851.06	336.75	200.49	-2.53	**0.012**
Sex	6869.73	1177.05	148.97	5.84	**<0.001**
Scanner	515.89	987.18	241.55	0.52	0.602
Psychotropic medication use	-1425.62	1529.09	234.82	-0.93	0.352

BDo = bipolar disorder offspring, Co = controls, NOS = number of streamlines, SZo = schizophrenia offspring.

[a]Linear mixed model analyses were run for each network metric with group, age, group*age, sex, scanner and psychotropic medication use (yes/no) as fixed effects, and subject and family as random effects.

[b]Analyses run with control offspring as reference group.

[c]Analyses run with bipolar disorder offspring as reference group.

Supplemental Table S17. Main effect of Age (representing the effect of age on network metric in Co) and Group*Age interaction effects for NOS-weighted global network metrics with presence of any lifetime psychiatric diagnosis (yes/no) added as covariate

Network metric[a]	*ß*	Standard error	*df*	*t*	*p*
Connectivity strength					
(Intercept)[b]	108805.33	2598.19	160.68	41.88	<0.001
Age in Co[b]	1104.39	462.87	181.43	2.39	0.018
Age-slope BDo vs Co[b]	-127.02	539.38	167.45	-0.24	0.814
Age-slope SZo vs Co[b]	-1492.54	613.70	178.56	-2.43	**0.016**
Age-slope SZo vs BDo[c]	-1365.52	557.15	197.61	-2.54	**0.015**
Sex	10503.76	1999.88	153.80	5.25	**<0.001**
Scanner	3127.34	1637.13	241.27	1.91	0.057
Lifetime psychiatric diagnosis	-229.42	2168.25	148.05	-0.11	0.916
Global efficiency					
(Intercept)[b]	47.67	1.14	160.45	41.77	<0.001
Age in Co[b]	0.54	0.20	182.54	2.61	**0.010**
Age-slope BDo vs Co[b]	-0.09	0.24	168.63	-0.39	0.700
Age-slope SZo vs Co[b]	-0.66	0.27	179.82	-2.44	**0.015**
Age-slope SZo vs BDo[c]	-0.57	0.25	198.99	-2.32	0.021
Sex	4.56	0.88	154.52	5.16	**<0.001**
Scanner	1.65	0.72	241.79	2.29	0.023
Lifetime psychiatric diagnosis	-0.26	0.96	148.98	-0.27	0.788
Clustering					
(Intercept)[b]	32.64	0.67	157.53	49.00	<0.001
Age in Co[b]	0.24	0.12	175.37	2.07	0.040
Age-slope BDo vs Co[b]	0.03	0.13	161.33	0.23	0.820
Age-slope SZo vs Co[b]	-0.42	0.15	172.00	-2.71	**0.007**
Age-slope SZo vs BDo[c]	-0.45	0.14	190.63	-3.20	**0.002**
Sex	3.07	0.51	148.79	6.01	**<0.001**
Scanner	0.83	0.41	237.69	2.02	0.044
Lifetime psychiatric diagnosis	-0.21	0.56	143.97	-0.37	0.709
Modularity (*ß* and standard error × 10^{-4})					
(Intercept)[b]	6078.15	32.49	132.69	187.08	<0.001
Age in Co[b]	-7.81	7.53	215.56	-1.04	0.301
Age-slope BDo vs Co[b]	15.64	9.06	225.50	1.73	0.086
Age-slope SZo vs Co[b]	3.03	10.01	220.77	0.30	0.763
Age-slope SZo vs BDo[c]	-12.61	8.99	242.62	-1.40	0.162
Sex	-8.13	27.50	158.49	-0.30	0.768
Scanner	-22.00	25.20	237.66	-0.87	0.384
Lifetime psychiatric diagnosis	-16.24	28.97	146.21	-0.56	0.576
Rich club connectivity					
(Intercept)[b]	13757.62	689.41	159.79	19.96	<0.001
Age in Co[b]	184.12	112.03	165.83	1.64	0.102
Age-slope BDo vs Co[b]	-50.42	130.19	151.21	-0.39	0.699
Age-slope SZo vs Co[b]	-265.35	148.26	161.48	-1.79	0.075
Age-slope SZo vs BDo[c]	-214.93	136.08	175.30	-1.58	0.116
Sex	648.01	560.67	164.81	1.16	0.249
Scanner	1530.00	409.00	225.81	3.74	**<0.001**
Lifetime psychiatric diagnosis	-281.54	608.12	164.43	-0.46	0.644
Feeder connectivity					
(Intercept)[b]	25687.37	846.35	154.54	30.35	<0.001
Age in Co[b]	250.51	168.19	196.86	1.49	0.138
Age-slope BDo vs Co[b]	-91.68	198.56	185.35	-0.46	0.645
Age-slope SZo vs Co[b]	-351.30	223.18	196.37	-1.57	0.117
Age-slope SZo vs BDo[c]	-259.62	201.95	217.45	-1.29	0.200
Sex	2973.95	688.78	160.62	4.32	**<0.001**
Scanner	1278.56	580.90	245.62	2.20	0.029
Lifetime psychiatric diagnosis	-201.57	739.46	155.59	-0.27	0.786
Local connectivity					
(Intercept)[b]	69373.57	1564.66	157.26	44.34	<0.001
Age in Co[b]	648.24	279.96	182.36	2.32	0.022
Age-slope BDo vs Co[b]	1.23	326.01	168.19	<0.01	0.997
Age-slope SZo vs Co[b]	-860.20	371.28	179.50	-2.32	0.022
Age-slope SZo vs BDo[c]	-861.42	336.51	199.09	-2.56	**0.011**
Sex	6830.30	1187.59	148.33	5.75	**<0.001**
Scanner	464.53	987.22	242.47	0.47	0.638
Lifetime psychiatric diagnosis	221.80	1287.52	141.70	0.17	0.863

BDo = bipolar disorder offspring, Co = controls, NOS = number of streamlines, SZo = schizophrenia offspring.

[a]Linear mixed model analyses were run for each network metric with group, age, group*age, sex, scanner and presence of any lifetime psychiatric diagnosis (yes/no) as fixed effects, and subject and family as random effects.

[b]Analyses run with control offspring as reference group.

[c]Analyses run with bipolar disorder offspring as reference group.

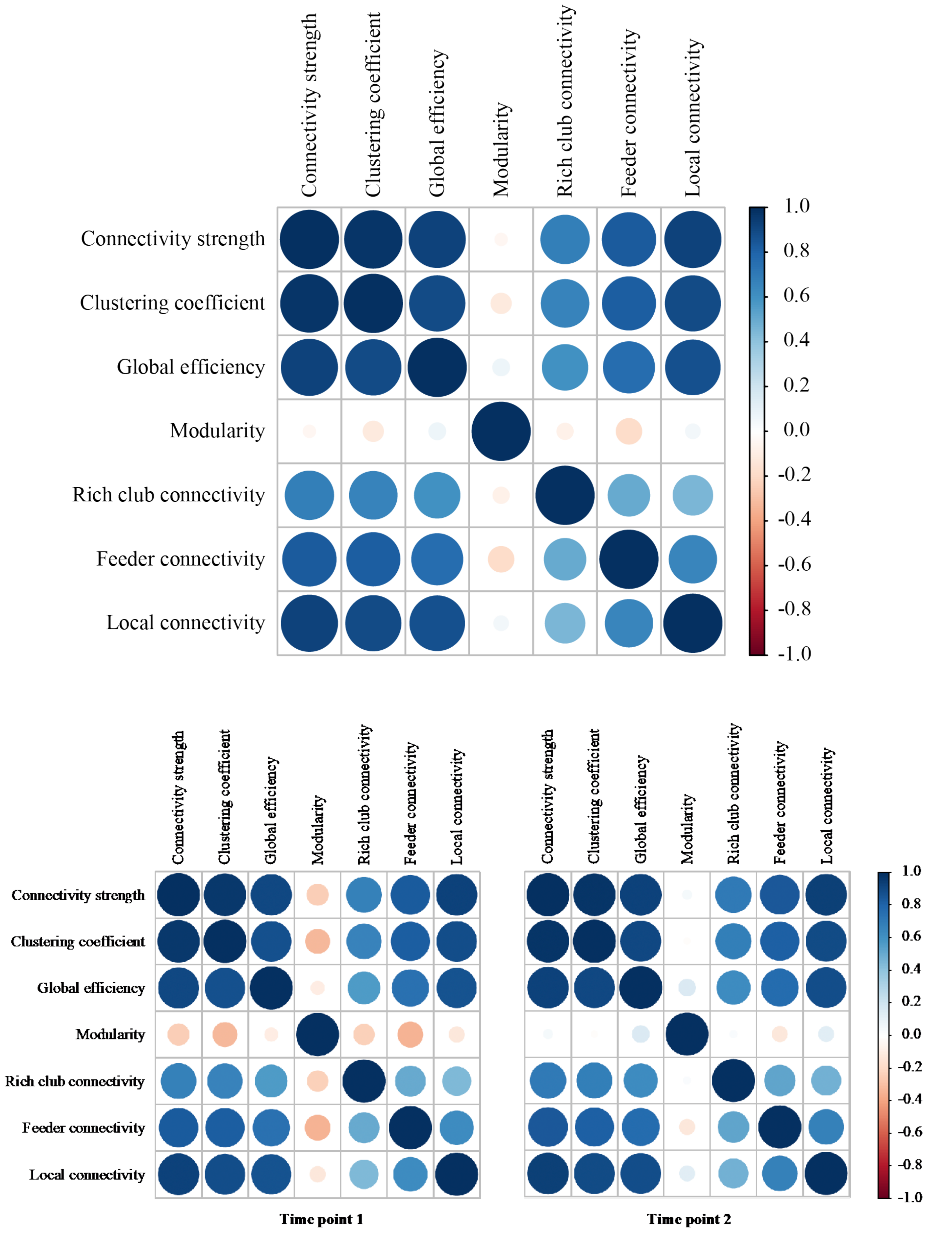

Supplemental Figure S1. Correlation matrices of the seven investigated NOS-weighted network metrics. (top) Entire dataset (values of participants who were scanned at both time points were averaged first); (bottom) Datasets at time point 1 (left) and time point 2 (right).

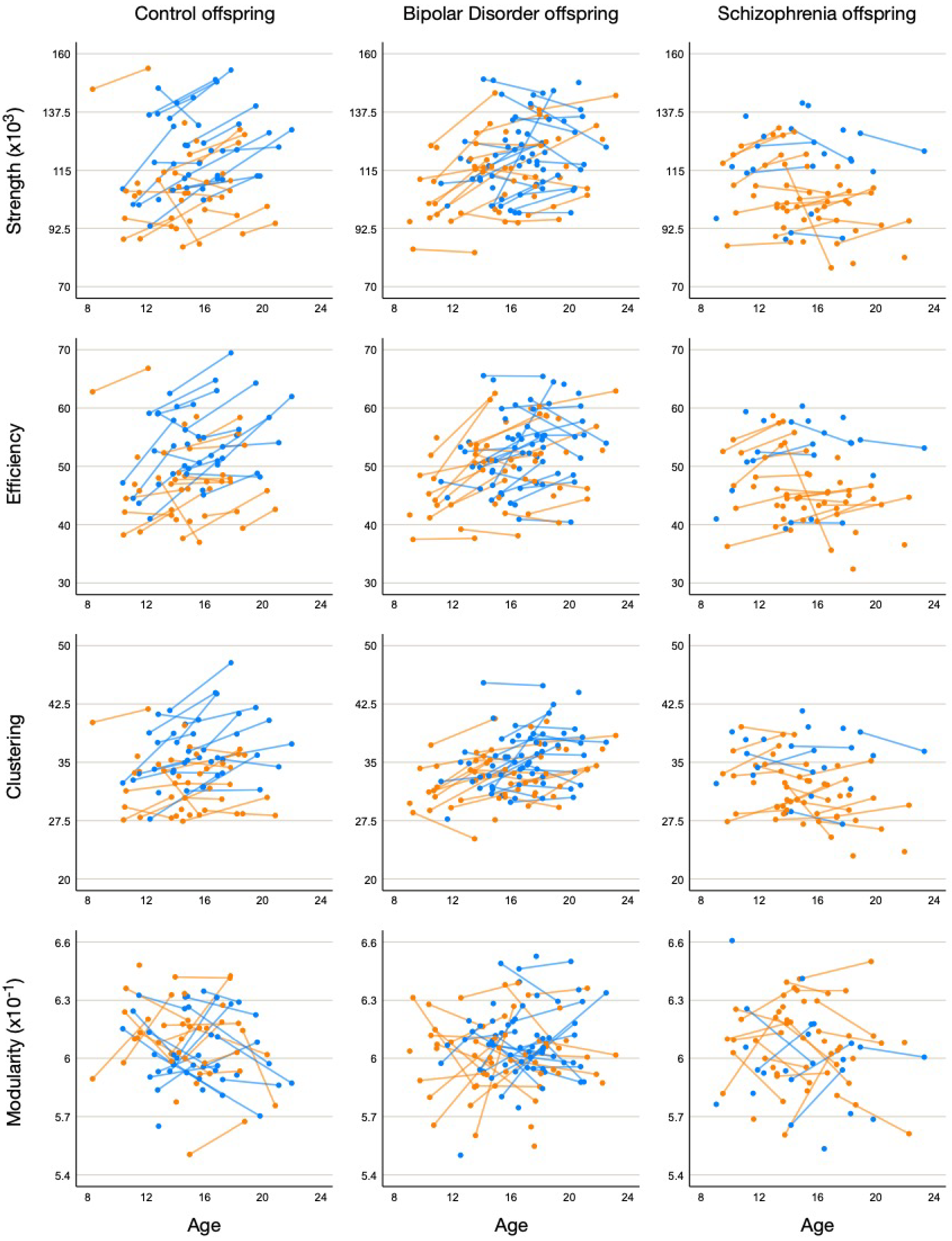

Supplemental Figure S2. Global network metrics as a function of age per group, with colors based on biological sex (orange = female; blue = male). Raw data points are presented with the model fit and standard error.

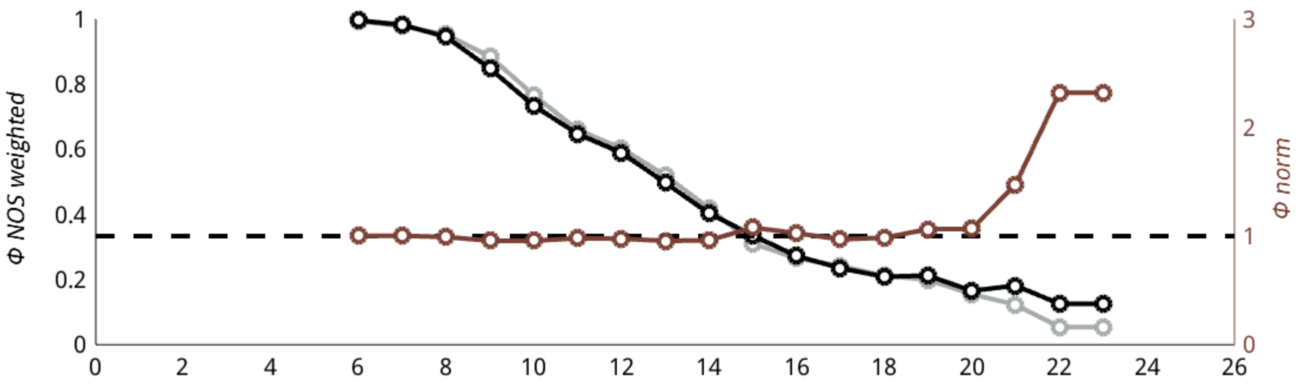

Supplemental Figure S3. Normalised rich club curve (relative to random rich club curve based on average 10000 random networks) for the weighted group-averaged network of all participants. A normalised coefficient Φ greater than 1 over a range of *k* suggests the existence of rich club organization in a network (34,35).

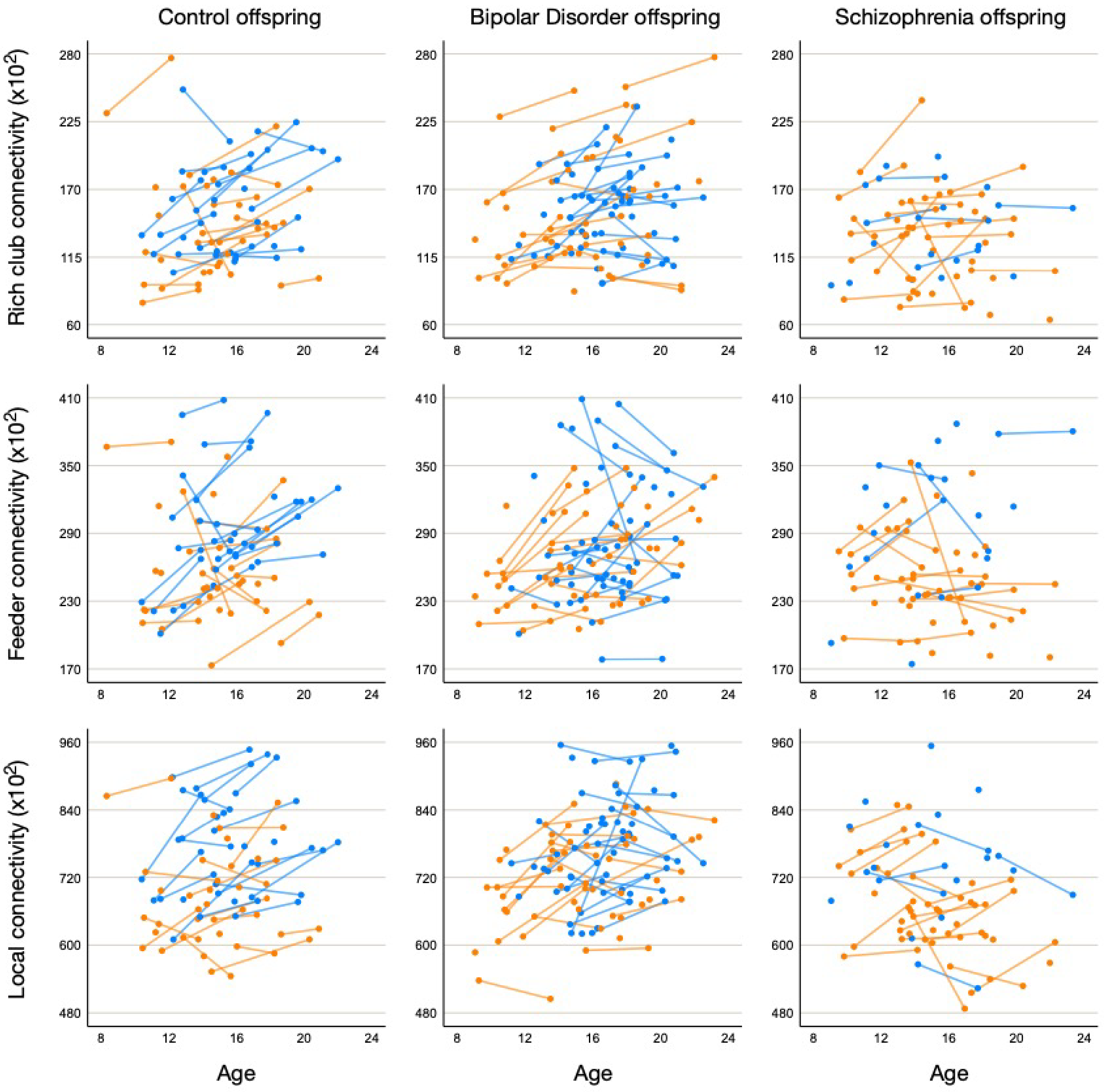

Supplemental Figure S4. Rich club, feeder and local connectivity as a function of age per group, with colors based on biological sex (orange = female; blue = male). Raw data points are presented with the model fit and standard error.

References

1. Kaufman J, Birmaher B, Brent D, Rao U, Ryan N (2000): Nederlandse vertaling van schedule of affective disorders and schizophrenia for school-age children—present and lifetime version (K-SADS-PL) (Reichart, Wals, & Hillegers, trans.). Utrecht, Netherlands: H.C. Rümke Groep.
2. Wechsler D (2005): *WISC-III Handleiding En Verantwoording (Kort et al., Trans.)*, 3rd ed. Amsterdam, Netherlands: Harcourt Test Publishers.
3. Wechsler D III (2004): *WAIS-III Nederlandstalige Bewerking Wechsler Adult Intelligence Scale: Afname En Scoringshandleiding (Trans.)*, 3rd ed. Amsterdam, Netherlands: Harcourt Test Publishers.
4. Bischoff-Grethe A, Ozyurt IB, Busa E, Quinn BT, Fennema-Notestine C, Clark CP, *et al.* (2007): A technique for the deidentification of structural brain MR images. *Hum Brain Mapp* 28: 892–903.
5. Fischl B (2012): FreeSurfer. *Neuroimage* 62: 774–781.
6. Jenkinson M, Beckmann CF, Behrens TEJ, Woolrich MW, Smith SM (2012): FSL. *Neuroimage* 62: 782–790.
7. Dale AM, Fischl B, Sereno MI (1999): Cortical surface-based analysis. I. Segmentation and surface reconstruction. *Neuroimage* 9: 179–194.
8. Fischl B, Sereno MI, Dale AM (1999): Cortical surface-based analysis. II: Inflation, flattening, and a surface-based coordinate system. *Neuroimage* 9: 195–207.
9. Fischl B, Salat DH, Busa E, Albert M, Dieterich M, Haselgrove C, *et al.* (2002): Whole brain segmentation: automated labeling of neuroanatomical structures in the human brain. *Neuron* 33: 341–355.
10. Rosas HD, Liu AK, Hersch S, Glessner M, Ferrante RJ, Salat DH, *et al.* (2002): Regional and progressive thinning of the cortical ribbon in Huntington's disease. *Neurology* 58: 695–701.

11. Andersson JLR, Skare S, Ashburner J (2003): How to correct susceptibility distortions in spin-echo echo-planar images: application to diffusion tensor imaging. *Neuroimage* 20: 870–888.
12. Andersson JLR, Sotiropoulos SN (2016): An integrated approach to correction for off-resonance effects and subject movement in diffusion MR imaging. *Neuroimage* 125: 1063–1078.
13. Andersson JLR, Graham MS, Zsoldos E, Sotiropoulos SN (2016): Incorporating outlier detection and replacement into a non-parametric framework for movement and distortion correction of diffusion MR images. *Neuroimage* 141: 556–572.
14. Andersson JLR, Graham MS, Drobnjak I, Zhang H, Filippini N, Bastiani M (2017): Towards a comprehensive framework for movement and distortion correction of diffusion MR images: Within volume movement. *Neuroimage* 152: 450–466.
15. Andersson JLR, Graham MS, Drobnjak I, Zhang H, Campbell J (2018): Susceptibility-induced distortion that varies due to motion: Correction in diffusion MR without acquiring additional data. *Neuroimage* 171: 277–295.
16. Leemans A, Jones DK (2009): The B-matrix must be rotated when correcting for subject motion in DTI data. *Magn Reson Med* 61: 1336–1349.
17. de Lange SC, Helwegen K, van den Heuvel MP (2023): Structural and functional connectivity reconstruction with CATO - A Connectivity Analysis TOolbox. *Neuroimage* 273: 120108.
18. Greve DN, Fischl B (2009): Accurate and robust brain image alignment using boundary-based registration. *Neuroimage* 48: 63–72.
19. Chang L-C, Jones DK, Pierpaoli C (2005): RESTORE: robust estimation of tensors by outlier rejection. *Magn Reson Med* 53: 1088–1095.
20. Chang L-C, Walker L, Pierpaoli C (2012): Informed RESTORE: A method for robust estimation of diffusion tensor from low redundancy datasets in the presence of physiological noise artifacts. *Magn Reson Med* 68: 1654–1663.

21. Mori S, Crain BJ, Chacko VP, van Zijl PC (1999): Three-dimensional tracking of axonal projections in the brain by magnetic resonance imaging. *Ann Neurol* 45: 265–269.

22. Maier-Hein KH, Neher PF, Houde J-C, Côté M-A, Garyfallidis E, Zhong J, *et al.* (2017): The challenge of mapping the human connectome based on diffusion tractography. *Nat Commun* 8: 1349.

23. de Reus MA, van den Heuvel MP (2013): Estimating false positives and negatives in brain networks. *Neuroimage* 70: 402–409.

24. Zalesky A, Fornito A, Cocchi L, Gollo LL, van den Heuvel MP, Breakspear M (2016): Connectome sensitivity or specificity: which is more important? *Neuroimage* 142: 407–420.

25. Sarwar T, Ramamohanarao K, Zalesky A (2019): Mapping connectomes with diffusion MRI: deterministic or probabilistic tractography? *Magn Reson Med* 81: 1368–1384.

26. Rubinov M, Sporns O (2010): Complex network measures of brain connectivity: uses and interpretations. *Neuroimage* 52: 1059–1069.

27. Newman MEJ (2006): Modularity and community structure in networks. *Proc Natl Acad Sci U S A* 103: 8577–8582.

28. Wierenga LM, Bos MGN, Schreuders E, Vd Kamp F, Peper JS, Tamnes CK, Crone EA (2018): Unraveling age, puberty and testosterone effects on subcortical brain development across adolescence. *Psychoneuroendocrinology* 91: 105–114.

29. Ingalhalikar M, Smith A, Parker D, Satterthwaite TD, Elliott MA, Ruparel K, *et al.* (2014): Sex differences in the structural connectome of the human brain. *Proc Natl Acad Sci U S A* 111: 823–828.

30. Tunç B, Solmaz B, Parker D, Satterthwaite TD, Elliott MA, Calkins ME, *et al.* (2016): Establishing a link between sex-related differences in the structural connectome and behaviour. *Philos Trans R Soc Lond B Biol Sci* 371: 20150111.

31. Salminen LE, Tubi MA, Bright J, Thomopoulos SI, Wieand A, Thompson PM (2022): Sex is a defining feature of neuroimaging phenotypes in major brain disorders. *Hum Brain Mapp* 43: 500–542.

32. van Haren NEM, Setiaman N, Koevoets MGJC, Baalbergen H, Kahn RS, Hillegers MHJ (2020): Brain structure, IQ, and psychopathology in young offspring of patients with schizophrenia or bipolar disorder. *Eur Psychiatry* 63: e5.

33. de Zwarte SMC, Brouwer RM, Agartz I, Alda M, Alonso-Lana S, Bearden CE, *et al.* (2022): Intelligence, educational attainment, and brain structure in those at familial high-risk for schizophrenia or bipolar disorder. *Hum Brain Mapp* 43: 414–430.

34. Uher R, Pavlova B, Radua J, Provenzani U, Najafi S, Fortea L, *et al.* (2023): Transdiagnostic risk of mental disorders in offspring of affected parents: a meta-analysis of family high-risk and registry studies. *World Psychiatry* 22: 433–448.

35. Collin G, Scholtens LH, Kahn RS, Hillegers MHJ, van den Heuvel MP (2017): Affected anatomical rich club and structural–functional coupling in young offspring of schizophrenia and bipolar disorder patients. *Biol Psychiatry* 82: 746–755.

36. Opsahl T, Colizza V, Panzarasa P, Ramasco JJ (2008): Prominence and control: the weighted rich-club effect. *Phys Rev Lett* 101: 168702.

37. Colizza V, Flammini A, Serrano MA, Vespignani A (2006): Detecting rich-club ordering in complex networks. *Nat Phys* 2: 110–115.

38. McAuley JJ, da Fontoura Costa L, Caetano TS (2007): Rich-club phenomenon across complex network hierarchies. *Appl Phys Lett* 91: 084103.
